# Cell-Nonautonomous Signaling of FOXO/DAF-16 to the Stem Cells of *Caenorhabditis elegans*

**DOI:** 10.1371/journal.pgen.1002836

**Published:** 2012-08-16

**Authors:** Wenjing Qi, Xu Huang, Elke Neumann-Haefelin, Ekkehard Schulze, Ralf Baumeister

**Affiliations:** 1 Faculty of Biology, Bioinformatics, and Molecular Genetics, Center for Biochemistry and Molecular Cell Research, Freiburg, Germany; 2 Faculty of Medicine, Center for Biochemistry and Molecular Cell Research, Freiburg, Germany; 3 Renal Division, University Hospital Freiburg, Freiburg, Germany; 4 Centre for Biological Signaling Studies (BIOSS), University of Freiburg, Freiburg, Germany; 5 FRIAS Freiburg Institute for Advanced Studies, Section Life Sciences (LIFENET), University of Freiburg, Freiburg, Germany; Stanford University Medical Center, United States of America

## Abstract

In *Caenorhabditis elegans* (*C. elegans*), the promotion of longevity by the transcription factor DAF-16 requires reduced insulin/IGF receptor (IIR) signaling or the ablation of the germline, although the reason for the negative impact of germ cells is unknown. FOXO/DAF-16 activity inhibits germline proliferation in both *daf-2* mutants and *gld-1* tumors. In contrast to its function as a germline tumor suppressor, we now provide evidence that somatic DAF-16 in the presence of IIR signaling can also result in tumorigenic activity, which counteracts robust lifespan extension. In contrast to the cell-autonomous IIR signaling, which is required for larval germline proliferation, activation of DAF-16 in the hypodermis results in hyperplasia of the germline and disruption of the surrounding basement membrane. SHC-1 adaptor protein and AKT-1 kinase antagonize, whereas AKT-2 and SGK-1 kinases promote, this cell-nonautonomous DAF-16 function. Our data suggest that a functional balance of DAF-16 activities in different tissues determines longevity and reveals a novel, cell-nonautonomous role of FOXO/DAF-16 to affect stem cells.

## Author Summary

Previous studies have shown that DAF–16/FOXO transcription factor promotes longevity and stress resistance and inhibits tumor progression in the absence of insulin signaling. Here we show that active DAF-16 in the epidermis can shorten lifespan by promoting a tumorous germline phenotype. In contrast to the known inhibitory effect of insulin signaling upon DAF-16, an active insulin and PI3K signaling are required for DAF-16–mediated signaling to the germline. In addition, AKT-1– and SHC-1–mediated JNK signaling antagonize AKT-2 and SGK-1 to affect the reproductive system. This is to our knowledge the first report about a detrimental effect of DAF-16 on lifespan. Furthermore it emphasizes that DAF-16 activity is highly dependent on the cellular context and communication between different tissues.

## Introduction

The forkhead box O (FOXO) subfamily of Forkhead transcription factors is conserved from *Caenorhabditis elegans* (*C. elegans*) to mammals [1]. Mammalian FOXO transcription factors consist of four members: FOXO1, FOXO3, FOXO4 and FOXO6, whereas only one homologue, DAF-16, is encoded in the *C. elegans* genome. DAF-16/FOXO proteins are inactivated by the insulin/IGF-1 signaling (IIS) through PI3K and the AGC kinases Akt/SGK which promote its cytosolic localization [2]–[7]. Starvation reduces IIS, resulting in nuclear localization and activation of DAF-16. Stress stimuli also result in nuclear translocation and activate FOXO via JNK and MST1 even in the presence of Akt [8], [9].

The Akt/FOXO signaling network acts as a critical control mechanism at the intersection between cancer and stem cell biology. FOXO proteins have been considered as tumor suppressors, because of their ability to induce DNA damage repair, cell cycle arrest and apoptosis [10], [11]. Consistently, loss of functional FOXO is associated with tumorigenesis in various organs [11]–[13]. On the other hand, FOXO proteins are required for the long-term maintenance of both normal and cancer stem cells. Mice with FOXO1, FOXO3 and FOXO4 triple knockout display a marked reduction of hematopoietic stem cells due to increased physiological oxidative stress [14]. In the cancer stem cells of chronic myeloid leukemia, FOXO3 is enriched in the nucleus and essential for maintaining these cancer stem cells [15].

In *C. elegans*, DAF-16 is a key regulator of development, longevity and stress response. Active DAF-16 resulting from reduced IIS leads to dauer formation, enhanced stress response and lifespan extension [16]–[18]. Recent studies reveal that the cross-talk between the reproductive system and somatic tissues plays an important role in development and aging of *C. elegans*. During larval development DAF-16 inhibits robust proliferation of the germline through both cell-autonomous (the germline) and -nonautonomous (the musculature) mechanism [19]. In adult animals intestinal and muscular DAF-16 slow down reproductive aging via oocyte and germline quality maintenance [20]. Activation of DAF-16 upon reduced IIS also inhibits tumor growth in the germline of *gld-1* mutants [21], indicating that the role of FOXO as tumor suppressor is evolutionarily conserved. On the other side, signals from the reproductive system regulate DAF-16 activity in the soma: Elimination of mitotic germ cells results in nuclear entry of intestinal DAF-16 and extends lifespan [22], [23]. Ablation of the somatic gonad precursors, however, abrogates the lifespan extension of the germline-ablated animals [23]. Even though several factors, such as KRI-1, TCER-1 and DAF-9, have been found to be involved in transduction of such signaling [24], [25], the details of the signaling mechanism are still not well known.

In a previous study we have shown that the *C. elegans* p52Shc homolog SHC-1 modulates DAF-16 activity through promoting its nuclear entry (Neumann-Haefelin et al., 2008). SHC-1 negatively regulates IIS by inhibition of the insulin/IGF receptor DAF-2. SHC-1 also associates with MEK-1, the mitogen-activated protein kinase kinase 7 (MAPKK7), to activate a *C. elegans* JNK homolog JNK-1, thus affecting stress response and longevity [9], [26]. SHC-1 and MEK-1 also mediate activation of an alternative *C. elegans* JNK homolog, KGB-1, upon heavy metal stress [27]. Shc-like proteins have been found in metazoan animals from nematodes to humans, suggesting their roles might also be conserved in evolution [28].

Here, we report a novel role of DAF-16 activity in epidermal cells affecting the reproductive system in a cell-nonautonomous manner, resulting in germline hyperplasia and disruption of the surrounding extracellular matrix of *C. elegans*. The adapter protein SHC-1 modulates both IIS and JNK pathways to antagonize this hypodermal function of DAF-16. Our finding reveals a new aspect of DAF-16 activity besides its well known roles in the regulation of longevity, stress response and dauer formation. Our data show that these two aspects affect longevity differently and indicate that the capability of DAF-16 to extend lifespan is dependent on the balance of these two opposing effects.

## Results

### Mitotic proliferation of the germline counteracts transgenic DAF-16–mediated longevity

SHC-1 positively regulates the activity of DAF-16 by both inhibiting DAF-2 and activating JNK signaling pathways [26]. *shc-1* mutant animals are generally healthy, grow at a normal rate, and produce normal numbers of offspring [26], [27]. However, they live about 25% shorter than wild type animals and this reduced lifespan is accompanied by cytoplasmic retention of DAF-16 [26].

**Figure 1:**
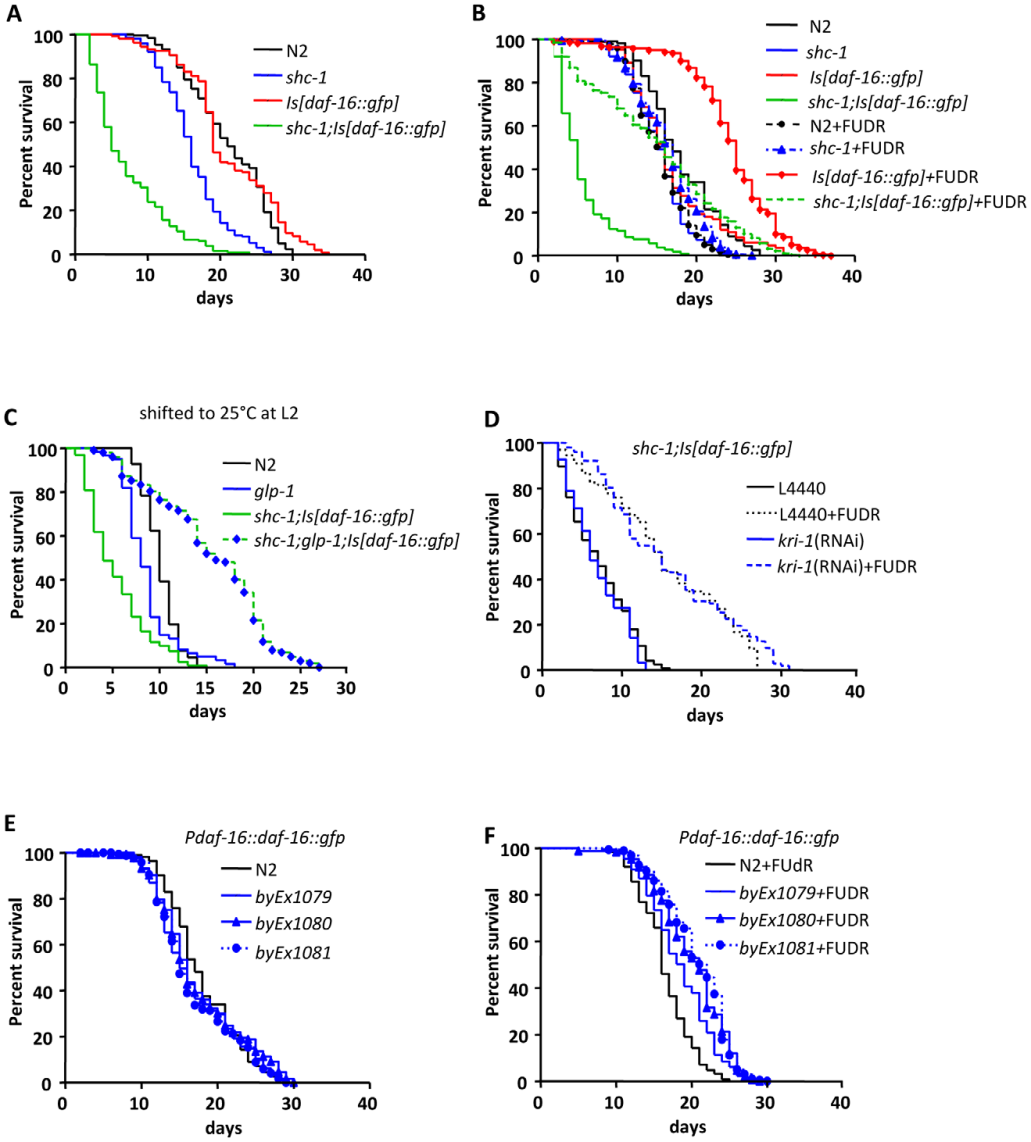
Lifespan of *daf-16* transgenic animals. (A) *shc-1(ok198);Is[daf-16::gfp]* animals show reduced lifespan. (B) Treatment with FUDR extends the lifespan of both *shc-1(ok198);Is[daf-16::gfp]* and *Is[daf-16::gfp]* animals. (C) Knock-down of *glp-1* extends lifespan of *shc-1(ok198);Is[daf-16::gfp]* animals (See also Figure S1, S2, S3 and Table S1). (D) The lifespan extension by FUDR does not require the adapter protein KRI-1. (E) In the absence of FUDR extrachromosomal *daf-16::gfp* transgene does not extend lifespan of wild type animals. (F) In the presence of FUDR extrachromosomal *daf-16::gfp* transgene extends lifespan of wild type animals. In the lifespan assay with FUDR treatment, animals were transferred onto the agar plates containing 0.1 mg/ml FUDR and *E.coli* 24 hours after the L4 larval stage. These were raised on the FUDR containing plates for four days and then transferred onto new plates without FUDR. The mean lifespan and statistical analyses in this figure are summarized in Table 1.

Since, according to this model, *daf-16* acts downstream of *shc-1*, access of wild type DAF-16, in particular the proportion that escapes phosphorylation by active AKT-1/AKT-2/SGK-1, should compensate for the loss of *shc-1* during the control of lifespan. We tested this hypothesis relying on the frequently used strain TJ356 which expresses the full length *daf-16* isoform a fused to GFP in a wild type background [29]. TJ356 animals have increased DAF-16 activity, however, displayed lifespan comparable to wild type (Figure 1A and Table 1), consistent with a previous report [29]. To our surprise, the *daf-16* transgene did not extend, but further reduced the already short lifespan of *shc-1* (Figure 1A and Table 1). Remarkably, about 50% of the *shc-1(ok198);Is[daf-16::gfp]* adult animals died within the first five days of adulthood. In addition, about half of the animals were sterile and the remaining fertile animals showed a strongly reduced brood size (49 *vs.* 348, Table S2). In order to exclude that this phenotype is allele-specific or caused by a background mutation linked to the *shc-1* locus, we crossed another allele of *shc-1*, *tm1729*, into the *Is[daf-16::gfp]* background and observed the same phenotype (Table 1 and Table S2). We conclude that both *daf-16* expression and loss of *shc-1* contribute to the observed phenotypes. This suggests that the combination of *daf-16* transgene and *shc-1* mutant results in a synergistic effect not previously reported in either TJ356 or *shc-1(ok198)*.

**Table 1:** Lifespan of mutant strains.

Genotype	Mean lifespan (days)	Number (n) of examined animals	P value
**Lifespan at 20°C**			
N2 wild type	21.0	184	
*shc-1(ok198)*	16.1	178	
*shc-1(tm1729)*	14.4	79	
*Is[daf-16::gfp]*	21.4	112	
*shc-1(ok198);Is[daf-16::gfp]*	6.9	145	<0.0001^a^
*shc-1(tm1729);Is[daf-16;;gfp]*	8.2	55	<0.0001^b^
*shc-1(ok198);Is[daf-16::gfp]* L4440 (control)	7.0	72	
*shc-1(ok198);Is[daf-16::gfp] daf-16*(RNAi)	11.9	72	<0.0001^c^
**Lifespan at 25°C** *			
N2 wild type	9.6	127	
*glp-1(q231)*	8.2	130	
*shc-1(ok198);Is[daf-16::gfp*]	5.0	139	
*glp-1(q231);shc-1(ok198);Is[daf-16::gfp]*	16.1	172	<0.0001^i^
**Lifespan at 15°C**			
N2 wild type	24.7	97	
*daf-2(e1370)*	33.8	103	
*shc-1(ok198);Is[daf-16::gfp]*	11.6	169	
*daf-2(e1370);shc-1(ok198);Is[daf-16::gfp]*	31.5	79	<0.0001^k^
**Lifespan (no FUDR) §**			
N2 wild type	17.6	159	
*shc-1(ok198)*	15.6	167	
*Is[daf-16::gfp]*	17.1	137	
*shc-1(ok198);Is[daf-16::gfp]*	5.2	171	
*shc-1(ok198);Is[daf-16::gfp]* L4440 (control)	7.2	123	
*shc-1(ok198) kri-1*(RNAi)*;Is[daf-16::gfp]*	6.9	92	
*mek-1(ks54)*	10.4	157	
*byEx1079/[Pdaf-16::daf-16::gfp]*	17.0	115	0.5083^d^
*byEx1080/[Pdaf-16::daf-16::gfp]*	17.6	133	0.6695^d^
*byEx1081/[Pdaf-16::daf-16::gfp]*	17.0	169	0.4638^d^
**Lifespan (with FUDR) §**			
N2 wild type	15.5	199	
*shc-1(ok198)*	16.5	156	
*Is[daf-16::gfp]*	24.4	214	<0.0001^e^
*shc-1(ok198);Is[daf-16::gfp]*	15.3	276	<0.0001^f^
*shc-1(ok198);Is[daf-16::gfp]* L4440 (control)	15.5	167	
*shc-1(ok198) kri-1*(RNAi)*;Is[daf-16::gfp]*	15.9	102	0.1676^j^
*mek-1(ks54)*	13.5	127	<0.0001^g^
*byEx1079/[Pdaf-16::daf-16::gfp]*	18.5	177	<0.0001^h^
*byEx1080/[Pdaf-16::daf-16::gfp]*	20.1	174	<0.0001^h^
*byEx1081/[Pdaf-16::daf-16::gfp]*	20.8	195	<0.0001^h^

P values relative to

a : *shc-1(ok198)*;

b : *shc-1(tm1729)*;

c : *shc-1(ok198);Is[daf-16::gfp]*+L4440;

d : N2 without FUDR;

e : *Is[daf-16::gfp]* without FUDR;

f : *shc-1(ok198);Is[daf-16::gfp]* without FUDR;

g : *mek-1(ks54)* without FUDR;

h : N2 with FUDR;

i : *shc-1(ok198);Is[daf-16::gfp]* from L2 at 25°C;

j : *shc-1(ok198);Is[daf-16::gfp]*+L4440 with FUDR;

k : *shc-1(ok198);Is[daf-16::gfp]* at 15°C.

* Animals were shifted to 25°C from L2 larval stage.

Unexpectedly, raising the adult animals in the presence of 5-fluoro-2′-deoxyuridine (FUDR), an inhibitor of DNA synthesis which blocks cell proliferation, suppressed the early lethality and extended the lifespan of *shc-1(ok198);Is[daf-16::gfp]* animals up to two fold (Figure 1B and Table 1). This was surprising, since no lifespan extension was observed upon FUDR treatment of wild type. FUDR is one of the most commonly used drugs in the treatment of colorectal cancer and frequently used in *C. elegans* lifespan assays to facilitate strain handling due to its ability to block cell proliferation and generation of progeny. Based on this result, we suggest that the substantial lifespan reduction and lethality of *shc-1(ok198);Is[daf-16::gfp]* may be linked to proliferation and, possibly, abnormal mitosis, and that this detrimental effect is blocked by FUDR.

The germline is the only tissue that undergoes mitosis in adult animals, so we asked whether mitosis of the germline leads to the early lethality of *shc-1(ok198);Is[daf-16::gfp]* animals. The GLP-1 mediated Notch signaling represses meiosis of the germ cells and keeps them in mitotic proliferation from the L3 larval stage. In *glp-1* mutants the germ cells stop mitotic cell division and enter meiosis [30]. If the lethality observed in *shc-1(ok198);Is[daf-16::gfp]* animals is indeed caused by proliferation of the germ cells, inactivation of *glp-1* should result in a similar lifespan extension as seen upon FUDR treatment. To test this assumption, we used the temperature sensitive allele *glp-1(q231)*, because *glp-1(q231)* animals at the restrictive temperature 25°C do not have a pronounced difference of mean lifespan compared to wild type probably due to a dysfunction in the atrophy intestine, in contrast to other long lived *glp-1(lf)* alleles, such as *e2141ts* or *q158* (Figure S1) [22], [25]. Therefore, alterations of lifespan caused by detrimental germline proliferation should be easily detectable in this stain. *shc-1(ok198);glp-1(q231);Is[daf-16::gfp]* animals lived 240% longer than *shc-1(ok198);Is[daf-16::gfp]* when shifted at the L2 larval stage to the restrictive temperature (Figure 1C and Table 1). *glp-1* mutation also extended lifespan of *shc-1;Is[daf-16::gfp]* animals when animals are re-shifted to 20°C after larval germline proliferation has ceased (Figure S2). In addition, the early lethality observed in *shc-1;Is[daf-16::gfp]* animals was completely abolished. Ablation of the mitotic germline or a *glp-1(e2141)* background extends lifespan of wild type animals, which require intestinal DAF-16 and the adaptor protein KRI-1. [23]. However, FUDR treatment did not extend lifespan of wild type animals (Figure 1B and Table 1), suggesting that FUDR uses a different mechanism as ablation of the germline to affect longevity of *shc-1;Is[daf-16::gfp]* animals. To validate this assumption, we further tested whether *kri-1* knock-down could suppress the FUDR dependent lifespan extension. *kri-1* RNAi treatment did not shorten the lifespan of *shc-1;Is[daf-16::gfp]* animals fed with FUDR (Figure 1D and Table 1). Based on these observations, we propose that early lethality of shc*-1(ok198);Is[daf-16::gfp]* animals is due to a negative input of germline proliferation rather than a general sickness of the strain.

We noticed in the strain TJ356 that transgenic *daf-16* extended lifespan of wild type animals significantly only in the presence of FUDR (Figure 1B). To further exclude any effect of the transgene used, we generated three independent lines carrying extra-chromasomal *daf-16::gfp* transgenes in wild type background. In the absence of FUDR, these strains also had lifespan like wild type animals (Figure 1E), whereas they lived 19.3%, 20.6%, and 26.7%, respectively, longer than wild type animals in the presence of FUDR (Figure 1F). Thus, transgenic DAF-16 indeed extends lifespan if germline proliferation is inhibited. Recently, FUDR has been shown to extend lifespan of *tub-1* mutant animals [31]. Together, this suggests that the use of FUDR in lifespan assays may be inconspicuous in some genetic backgrounds [32], but may strongly affect others.

### Transgenic expression of *daf-16* in *shc-1* mutant animals causes pleiotropic germline and gonad development defects

**Figure 2:**
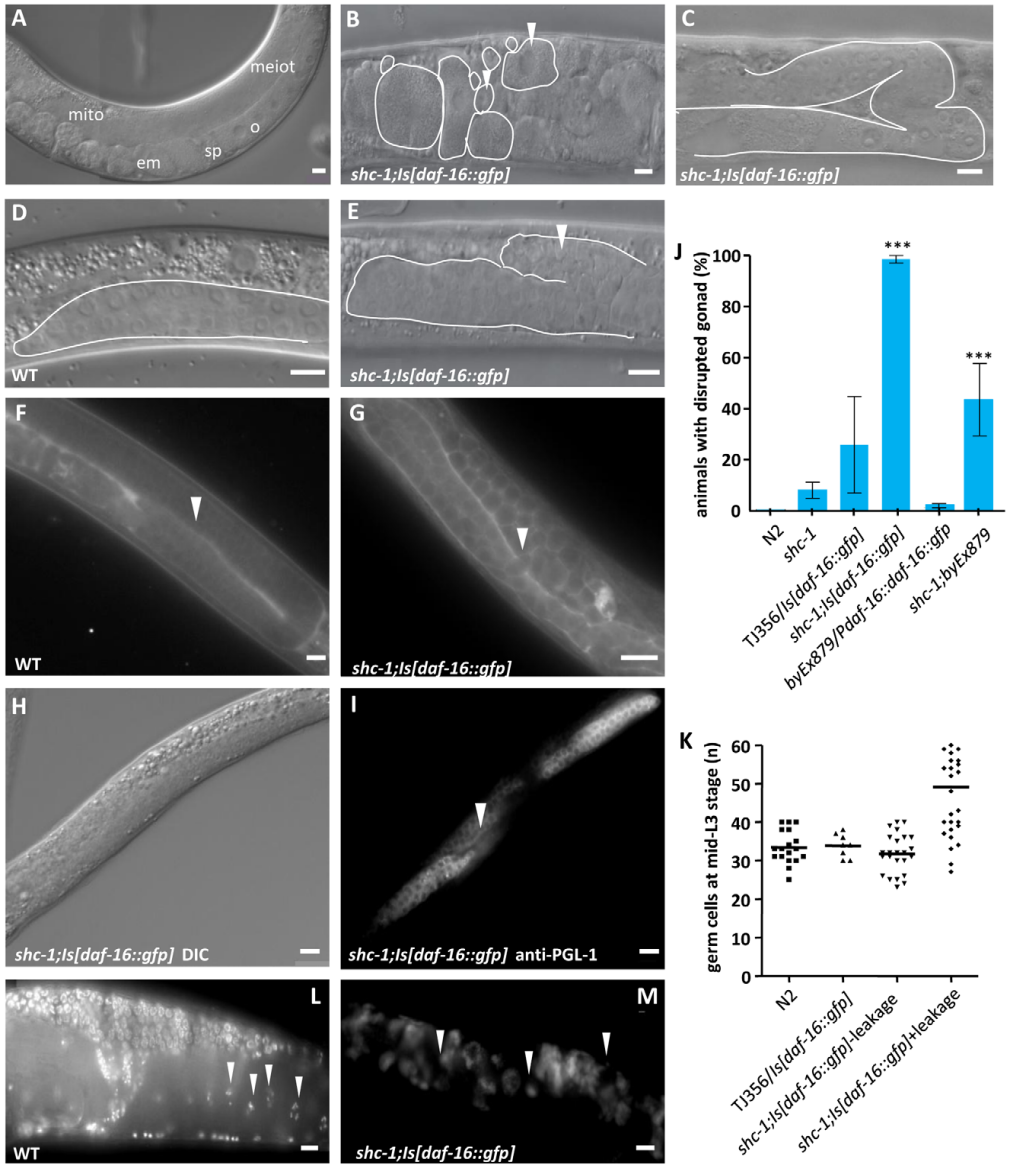
*shc-1(ok198);Is[daf-16::gfp]* animals display pleiotropic defects in the reproductive system. (A–C) DIC images of the gonad of one day adult animals. The white arrowhead denotes accumulated germ cells in the pseudocoelom (B) and a DTC migration defect (C). mito: mitotic region of the germline; meiot: meiotic region of the germline; o: oocyte; sp: sperm; em: embryo. (D and E) DIC images of the whole anterior gonad arm of L3 larvae. The white arrowhead denotes the germ cells leaking out from the gonad (E). (F and G) MitoTracker Red staining of the gonadal basement membrane. The white arrowhead points to the intact basement membrane in wild type (F) and disrupted basement membrane in *shc-1(ok198);Is[daf-16::gfp]* (G) animals. (H and I) DIC image (H) and anti-PGL-1 antibody staining (I) of *shc-1(ok198);Is[daf-16::gfp]* L3 larva. The white arrowhead indicates the anterior gonad with leakage (I). (J) Knock-down of *shc-1* gene enhances the defect in the basement membrane of animals carrying *daf-16* transgene. In this and the following figures data of defect in the basement membrane are presented as mean±SD; the mean values and statistic analysis are summarized in Table 2 and Table 3; the symbol * means P<0.01, ** means P<0.001 and *** means P<0.0001. (K) The gonad arms with disrupted basement membrane of *shc-1(ok198);Is[daf-16::gfp]* animals contain more germ cells than those with intact basement membrane at mid-L3 larval stage. For quantitative assessments PGL-1 antibody staining was performed to label the germ cells. Numbers of germ cells per gonad ±SD: wild type N2: 33±4 (n = 18); *Is[daf-16::gfp]*: 34±3 (n = 8); intact gonad of *shc-1;Is[daf-16::gfp]* animals: 32±5 (n = 24); disrupted gonad of *shc-1;Is[daf-16::gfp]* animals: 49±13 (n = 29) (P<0.0001 compared to N2, *Is[daf-16::gfp]* or intact gonad of *shc-1;Is[daf-16::gfp]* animals). (L and M) DAPI staining of wild type (L) and *shc-1(ok198);Is[daf-16::gfp]* (M) one day adult animals. The white arrowheads point to the chromosomes in the diakinetic oocytes of wild type animals and DNA in the endomitotic germ cells of *shc-1(ok198);Is[daf-16::gfp]* animals, respectively. Scale bar 10 µm.

Analysis of *shc-1(ok198);Is[daf-16::gfp]* animals using DIC microscopy revealed that the reproductive system exhibited multiple defects (Figure 2). The most prominent phenotype was the accumulation of cells in the pseudocoelom in almost all of the one day old adult animals (Figure 2B, 2J and Figure S3). These cells superficially looked like germ cells, yet were clearly localized outside the gonad. In the background of either *glp-1(q231)* or *glp-1(e2141)* these extra cells were absent, indicating that they may be germ cells (Figure S3). They showed substantial variations of shape and size. In some animals the boundary of the gonad was totally disrupted. It was, therefore, not possible to extract the gonad of these animals in an intact form (data not shown). In gonads with still recognizable morphology, a defect in the distal tip cell (DTC) migration was observed (Figure 2C and Figure S4). Analysis of *shc-1(ok198);Is[daf-16::gfp]* at different larval stages revealed that the gonad ruptured at the L3 larval stage at the proximal side next to the developing somatic gonad primordium. In animals in which germ cells leaked out into the pseudocoelom, a sharp gonad boundary indicative of an intact gonadal basement membrane, as seen in the wild type animals (Figure 2D), was absent in *shc-1(ok198);Is[daf-16::gfp]* animals (Figure 2E and Figure S5). The gonadal basement membrane can be visualized by staining with MitoTracker [33], and MitoTracker staining confirmed its partial disruption and leakage (Figure 2G and Figure S5).

In order to determine the identity of the extra-gonadal cells, we stained *shc-1(ok198);Is[daf-16::gfp]* animals with an antibody against the germ cell specific protein PGL-1, a P-granule component. The released cells were PGL-1 positive, corroborating their identity as germ cells (Figure 2I, Movie S1). We noticed that at the mid-L3 stage the disrupted gonad arms of *shc-1(ok198);Is[daf-16::gfp]* animals contained significantly more germ cells than in wild type (46±14 *vs.* 35±4) (Figure 2K). In *shc-1(ok198);Is[daf-16::gfp]* animals in which one gonad arm was still intact, we counted more germ cells in the disrupted gonad arm than in the intact one. In adult animals the volume of some of these released cells increased so that they looked like oocytes. However, they lacked the condensed diakinetic chromosomes that are characteristic for oocytes (Figure 2L). Instead, some of them displayed endomitotic chromosomes (Figure 2M). Taking together, these data suggest that *shc-1(ok198);Is[daf-16::gfp]* animals show abnormal larval germline proliferation and disruption of the gonadal basement membrane. In the following results we focus mainly on the disruption of gonad phenotype.

### Transgenic DAF-16 enhances a weakly penetrant phenotype present in *shc-1(ok198)*

**Figure 3:**
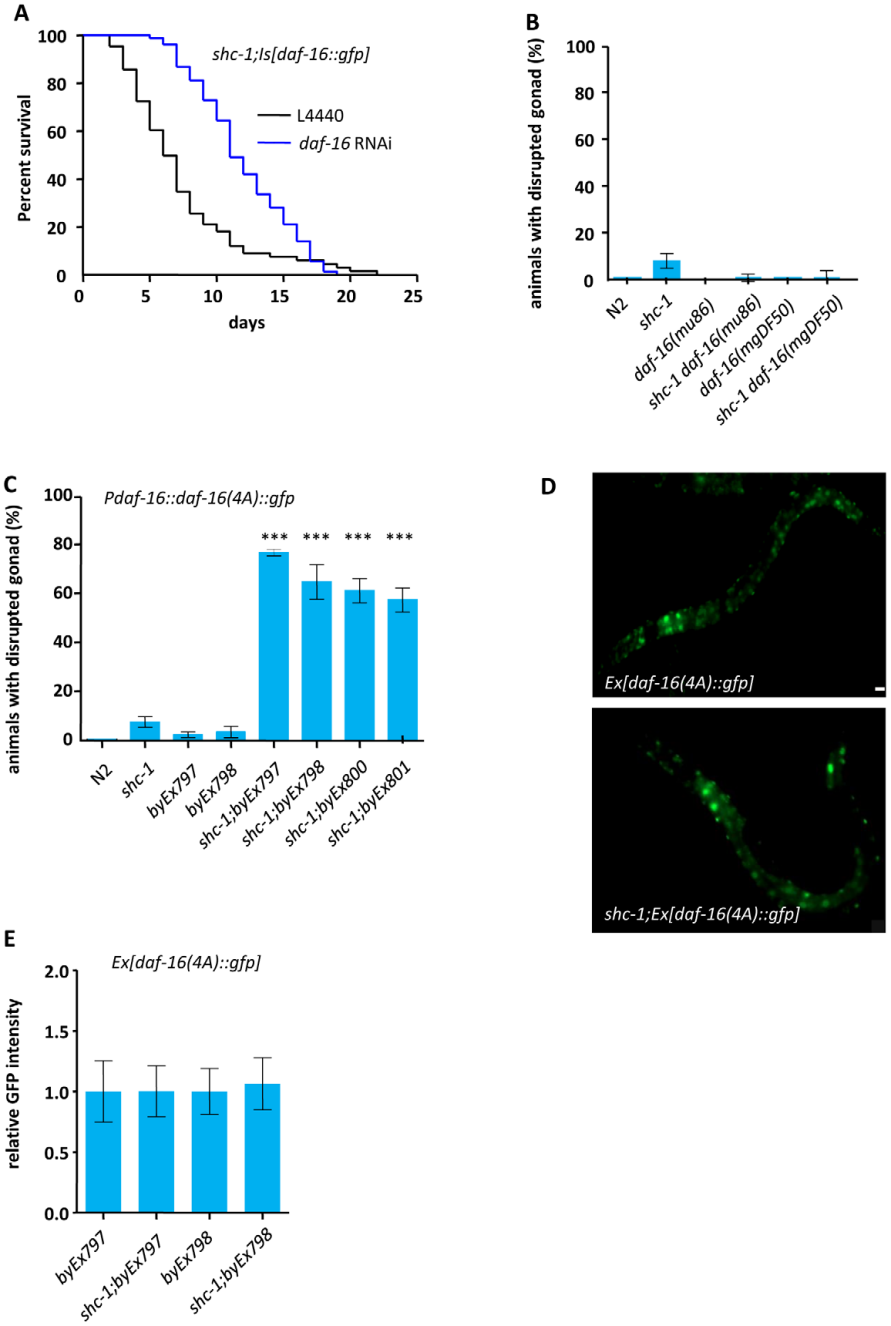
Active DAF-16 derogates integrity of the gonadal basement membrane. (A) Knock-down of *daf-16* by RNAi suppressed the early lethality of shc*-1(ok198);Is[daf-16::gfp]* animals. Animals died within the first five days of adulthood: *shc-1(ok198);Is[daf-16::gfp](L4440)* as control: 45.7%, *shc-1(ok198);Is[daf-16::gfp]+daf-16* RNAi: 1.4%. (B) *daf-16* mutation suppresses the defects in gonad integrity of *shc-1* mutant animals. (C) Transgenic expression of a constitutively nuclearly located *daf-16(4A)::gfp* results in disruption of the gonad in *shc-1(−)*, but not in wild type animals (See also Figure S7 and Table 3). (D) SHC-1 does not affect the subcellular localization of DAF-16(4A)::GFP. In both wild type and *shc-1(ok198)* animals DAF-16(4A)::GFP is constitutively located in the nucleus. The mean lifespan and statistical analyses in this figure are summarized in Table 1. *byEx* represents extrachromosomal transgenic alleles which are summarized in Table 3. Mean values and statistic analysis of defect in gonadal integrity are summarized in Table 2 and Table 3. (E) SHC-1 does not affect expression level of transgenic *daf-16(4A)::gfp*. Columns represent pooled normalised values of three independent experiments plus standard deviation (SD). Mann Whitney test. Normalized GFP intensity: *byEx797*: 1.00±2.5 (n = 31); *shc-1;byEx797*: 1.00±2.1 (n = 33), P = 0.9670; *byEx798*: 1.00±0.19 (n = 37); *shc-1;byEx798*:1.06±0.22 (n = 48), P = 0.1591.

Knock-down of *daf-16* expression by RNAi significantly suppressed all phenotypic aspects (low brood size, sterility, gonad disruption and early adult lethality) of *shc-1(ok198);Is[daf-16::gfp]* animals (Figure 3A, Table 1 and Table S2). The suppression was only partial since knock-down of *daf-16* by RNAi feeding was incomplete, indicated by a weak but persistent expression of DAF-16::GFP (Figure S6).

In order to rule out an involvement of background mutations, we generated new transgenic animals carrying extra-chromosomal *daf-16::gfp* in *shc-1* mutants. These strains displayed similar but less severe germline and gonad defects (Figure 2J and Table 3), which is probably due to the weaker DAF-16::GFP expression in extrachromosomal *vs.* integrated line (Figure S7) or due to the mosaic inheritance of extra-chromosomal transgenes.

The phenotype we observed in *shc-1;Is[daf-16::gfp]* animals was stronger than in *Is[daf-16::gfp]* animals, suggesting that reduced SHC-1 activity enhances DAF-16. This is in apparent contrast to our previous experiments in which we showed that DAF-16 activation is decreased in *shc-1* mutant [26]. To understand this obvious discrepancy, we tested whether disruption of gonad phenotype could also be caused by inactivating DAF-16 in either wild type or *shc-1(ok198)* mutant background. No gonad disruption was seen in either *daf-16(mu86)* or *daf-16(mgDF50)* animals (Figure 3B and Table 2), suggesting that reduction of *daf-16* activity was not the cause of this defect. However, about 7% of the *shc-1(ok198)* animals showed gonad disruption, yet with a delayed onset. Deletion of *daf-16* almost fully suppressed this defect in s*hc-1(ok198)* animals (Figure 3B and Table 2). This strongly suggests that the germline phenotype is not a synthetic or artificial phenotype seen only in *shc-1;Is[daf-16::gfp]* animals, and that DAF-16 and SHC-1 affect the reproductive system of *C. elegans* in opposite way.

**Table 2:** Defects in gonadal integrity.

Genotype	% one day adult with disrupted gonad		
	Mean±SD	n	P Value
N2 wild type	0	150	
*shc-1(ok198)*	7.3±4.9	120	<0.0001^a^
*Is[daf-16::gfp]*	25.8±18.9	240	<0.0001^a^
*shc-1(ok198);Is[daf-16::gfp]*	98.5±2.9	120	<0.0001^a^^,^ b
*daf-16(mu86)*	0	150	
*shc-1(ok198) daf-16(mu86)*	0.7±0.7	150	0.4226^a^
*daf-16(mgDF50)*	0	90	
*shc-1(ok198) daf-16(mgDF50)*	1.2±1.6	120	0.6486^a^
*shc-1(ok198) daf-16(mu86);Is[daf-16::gfp]*	97.8±1.9	90	0.9998^c^
**IIS pathway**			
*daf-2(e1370)*	0	120	
*shc-1(ok198);daf-2(e1370)*	6.7±6.7	90	0.8433^e^
*shc-1(ok198);Is[daf-16::gfp]*	100	120	
*shc-1(ok198);daf-2(e1370);Is[daf-16::gfp]*	53.6±1.9#	90	<0.0001^l^
*akt-1(ok525)*	0	120	
*shc-1(ok198);akt-1(ok525)*	58.5±21.0	217	<0.0001^e^
*akt-2(ok393)*	0	120	
*shc-1(ok198);akt-2(ok393)*	4.2±1.9	120	0.3501^e^
N2 *sgk-1*(RNAi)	0	120	
*shc-1(ok198);sgk-1*(RNAi)	9.2±6.9	120	0.3702^f^
*shc-1(ok198);akt-1(ok525)* #	59.3±14.0	120	
*daf-2(e1370);shc-1(ok198);akt-1(ok525)* #	2.2±1.9	90	<0.0001^g^
*daf-18(e1375)*	4.2±1.7	120	
*daf-18(ok480)*	0.9±0.7	96	
*daf-18(e1375);akt-1(ok525)*	5.0±4.3	120	0.1205^h^
*shc-1(ok198);daf-18(e1375);akt-1(ok525)*	93.6±5.7	120	<0.0001^i^
*shc-1(ok198) daf-16(mu86);daf-18(e1375);akt-1(ok525)*	4.4±2.0	90	<0.0001^j^
*shc-1(ok198);akt-1(ok525)* (L4440)	50.6±14.4	180	
*shc-1(ok198);akt-1(ok525);akt-2*(RNAi)	16.0±11.4	150	0.0178^k^
*shc-1(ok198);akt-1(ok525);sgk-1*(RNAi)	16.7±8.2	120	0.0029^k^
**JNK pathway**			
*mek-1(ks54)*	0	90	
*jnk-1(kg7)*	0	120	
*kgb-1(RNAi)*	4.2±3.2	120	
*mek-1(ks54);Is[daf-16::gfp]*	95.6±5.1	90	<0.0001^b^
*jnk-1(kg7) Is[daf-16::gfp]*	16.7±6.1	120	0.3757^b^
*Is[daf-16::gfp]* (L4440)	34.2±16.4	120	
*kgb-1*(RNAi) *Is[daf-16::gfp]*	73.3±8.8	90	<0.0001^d^

n: Numbers of examined animals.

# : Animals were raised at 15°C until examination.

P values were relative to:

a : N2;

b : *Is[daf-16::gfp]*;

c : *shc-1(ok198);Is[daf-16::gfp]*;

d : *Is[daf-16::gfp]*(L4440);

e : *shc-1(ok198)*;

f : *shc-1(ok198)*(L4440);

g : *shc-1(ok198);akt-1(ok525)* #;

h : *akt-1(ok525)*;

i : *shc-1(ok198);akt-1(ok525)*;

j : *shc-1(ok198);daf-18(e1375);akt-1(ok525)*;

k : *shc-1(ok198);akt-1(ok525)*;

l : *shc-1(ok198);Is[daf-16::gfp]* at 15°C.

### Nuclear DAF-16 signals to the germline

Since we have previously shown that SHC-1 promotes nuclear localization of DAF-16 by inhibiting DAF-2 and activating JNK-1, we expected an increase of cytosolic *vs.* nuclear DAF-16::GFP in *shc-1(ok198);Is[daf-16::gfp]* compared to *Is[daf-16::gfp]* animals [26]. If increased cytosolic *vs.* nuclear DAF-16::GFP would be the cause of the observed phenotype, then expression of a constitutively nuclear *daf-16* mutant (*daf-16(4A)::gfp*) should not result in gonad disruption. Expressing *daf-16(4A)::gfp* in wild type animals caused only weak disrupted gonad phenotype (Figure 3C and Table 3). However, transgenic expression of *daf-16(4A)::gfp* in a *shc-1(−)* background caused severe defects. We observed that DAF-16(4A)::GFP was nuclearly localized and this nuclear localization was not affected by either presence or absence of SHC-1 (Figure 3D). In addition, *shc-1* mutation did not affect the expression level of *daf-16(4A)::gfp* (Figure 3E). We also compared GFP intensities in the *daf-16::gfp* strains and correlated them to the severity of the phenotype. We found that, to result in a comparable phenotype, wild type *daf-16::gfp* required a higher expression level than *daf-16(4A)::gfp* (Figure S7). Given that wild type DAF-16 is mostly retained in the cytoplasm, this observation also indicates that nuclear instead of cytosolic DAF-16::GFP is the cause for the phenotype and SHC-1 antagonizes DAF-16 to ensure the gonadal integrity not simply via affecting its subcellular localization.

**Table 3:** Gonad basement membrane phenotype of animals with transgenic *daf-16::gfp* or *daf-16(4A)::gfp*.

Genotype	Transgene	Concentration injected (ng/µl)	% one day adult with disrupted gonad	
			Mean±SD	P-Value
N2	none*		0	
*shc-1(ok198)*	none*		6.1±2.7	
N2	*Pdaf-16::daf-16::gfp/byEx879*	100	1.1±1.9	0.2866^a^
N2	*byEx1079*	100	n.d	
N2	*byEx1080*	100	n.d	
N2	*byEx1081*	100	n.d	
N2	*Pdaf-16::daf-16(4A)::gfp/byEx797*	50	2.2±1.9	0.1165^a^
N2	*byEx798*	50	3.3±4.7	0.2871^a^
*shc-1(ok198)*	*Pdaf-16::daf-16::gfp/byEx879*	100	45.5±10.7	<0.0001^b^^,^ c
*shc-1(ok198)*	*Pdaf-16::daf-16(4A)::gfp/byEx797*	50	76.7±2.7	<0.0001^b^^,^ d
*shc-1(ok198)*	*byEx798*	50	68.7±14.5	<0.0001^b^^,^ d
*shc-1(ok198)*	*byEx800*	50	61.1±1.2	<0.0001^b^^,^ d
*shc-1(ok198)*	*byEx801*	50	57.2±9.9	<0.0001^b^^,^ d
*shc-1(ok198)*	*Pmyo-3::daf-16(4A)::gfp/byEx862*	50	4.4±7.7	0.5310^b^
*shc-1(ok198)*	*/byEx863*	50	3.4±7.8	0.2654^b^
*shc-1(ok198)*	*Pges-1::daf-16(4A)::gfp/byEx865*	50	6.6±5.8	0.8692^b^
*shc-1(ok198)*	*byEx866*	50	7.8±5.1	0.9105^b^
*shc-1(ok198)*	*Punc-119::daf-16(4A)::gfp/byEx895*	50	1.1±1.9	0.2654^b^
*shc-1(ok198)*	*byEx896*	50	3.3±3.4	0.6436^b^
*shc-1(ok198)*	*Pdpy-7::daf-16(4A)::gfp/byEx920*	50	80.0±6.7	<0.0001^b^
*shc-1(ok198)*	*byEx921*	50	83.3±12.0	<0.0001^b^

Transgenes were coinjected with *rol-6*(pRF4) at 20 ng/µl.

* : The animals carry only the injection marker *rol-6*.

The P-values were relative to:

a N2,

b *shc-1(ok198)*,

c *Ex[daf-16::gfp]*,

d *Ex[daf-16(4A)::gfp]*.

n.d: not determined.

### AKT-1 ensures gonad integrity by inactivating DAF-16

**Figure 4:**
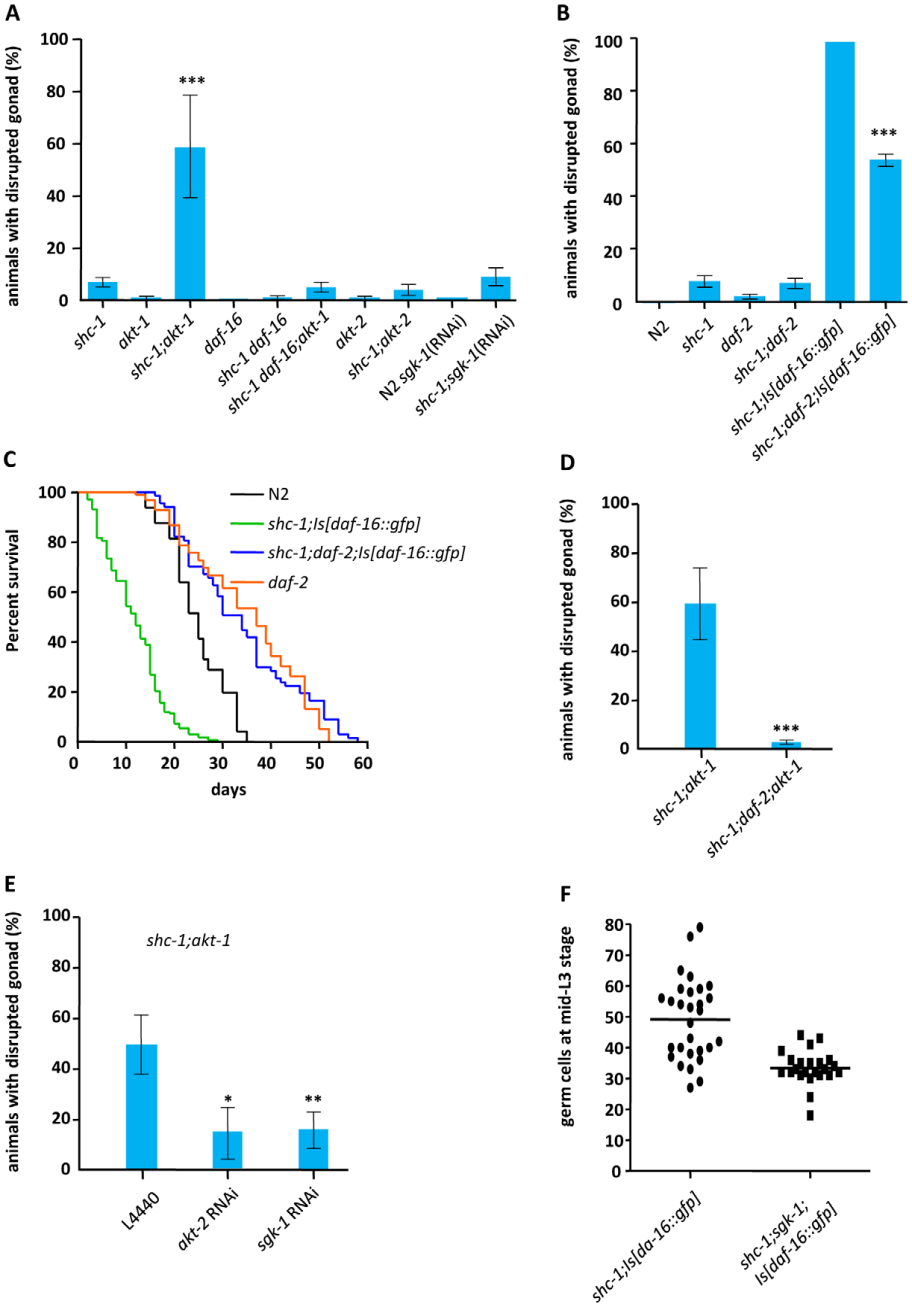
Active DAF-2, AKT-2 and SGK-1 antagonize AKT-1 mediated inhibition of DAF-16 to derogate gonad integrity. (A) DAF-16 activity affecting gonad integrity is inhibited by AKT-1. (B) Inactivation of IIR DAF-2 suppresses the defect in the gonadal basement membrane (See also Figure S8 and Table S2). (C) Inactivation of *daf-2* extends lifespan of *shc-1(ok198);Is[daf-16::gfp]* animals. (D) *daf-2* mutation suppresses gonad disruption in *akt-1;shc-1* animals (See also Figure S9). (E) AKT-2 and SGK-1 antagonize AKT-1. (F) *shc-1(ok198);sgk-1(ok538);Is[daf-16::gfp]* L3 larvea have less germ cells than *shc-1(ok198);Is[daf-16::gfp]* L3 larvea. *shc-1;Is[daf-16::gfp]* animals: 49±13 (n = 29); *shc-1;sgk-1;Is[daf-16::gfp]* animals: 33±6 (n = 23) (P<0.0001). (See also Figure S10). The mean lifespan and statistical analyses in this figure are summarized in Table 1. Mean values and statistic analysis of gonad disruption are summarized in Table 2.

AKT-1, AKT-2 and SGK-1 are known to phosphorylate DAF-16 directly and inhibit its nuclear entry [4], [7]. To test whether mutations in *akt-1*, *akt-2* or *sgk-1* that activate DAF-16 also influence the integrity of the gonadal basement membrane, *shc-1(ok198);akt-1(ok525)*, *shc-1(ok198);akt-2(ok393)* and *shc-1(ok198);sgk-1*(RNAi) were analyzed (Figure 4A and Table 2). Neither *akt-1(ok525)*, *akt-2(ok393)* single mutants nor *sgk-1*(RNAi) displayed gonad disruption. Inactivation of *akt-2* or *sgk-1* did not enhance the penetrance of the gonad defect of *shc-1(ok198)* animals, either. In contrast, 58.5±21.0% of first-day adult *shc-1(ok198);akt-1(ok525)* animals showed gonad disruption, providing further evidence that this phenotype is not an artificial effect of transgenic *daf-16* since this strain does not harbor a *daf-16* transgene. In addition, a loss-of-function allele of *daf-16* suppressed this phenotype in *shc-1;akt-1* animals, verifying its dependence on DAF-16 (Figure 4A). Taken together, these results strongly suggest that AKT-1 ensures gonadal integrity via inhibiting DAF-16.

### Active IIR DAF-2 contributes to gonad disruption defect caused by DAF-16

Since *akt-1* is downstream of *daf-2*, *shc-1;daf-2* double mutant should therefore exhibit the same phenotype as *akt-1;shc-1*. To test this, we crossed the temperature sensitive mutant *daf-2(e1370)* into *shc-1(ok198)* background. At 25°C both *daf-2(e1370)* and *shc-1(ok198);daf-2(e1370)* mutants formed constitutive dauer larvae with a developmentally arrested germline that prevented the analysis of adult animals. At 20°C, at which *daf-2* is partially inactivated, *daf-2(e1370)* did not alter the defect in the gonadal integrity in *shc-1* mutant (Figure 4B and Table 2).

In order to explore whether DAF-2 inhibits a DAF-16 dependent effect on the germline, we crossed *daf-2(e1370)* into *shc-1(ok198);Is[daf-16::gfp]* animals and quantified the percentage of animals displaying disrupted gonad at 15°C, since at 20°C *shc-1(ok198);daf-2(e1370);Is[daf-16::gfp]* showed development arrest due to excessive *daf-16* transgene expression (Figure S8). At 15°C, activity of DAF-2 in the *daf-2(e1370)* mutant is already reduced, indicated by an extended lifespan [34]. Surprisingly, *daf-2(e1370)* significantly reduced instead of increased the penetrance of animals with gonad disruption (Figure 4B and Table 2), indicating that this phenotype develops only in animals with intact IIS. In addition, the sterile phenotype of *shc-1(ok198);Is[daf-16::gfp]* animals was completely suppressed by *daf-2* mutation and the brood size of the fertile animals was increased (Table S2). Consistent with the suppression of the defects in the reproductive system, the early lethality in adulthood was abolished and lifespan was extended from 11.6 days to 31.5 days (Figure 4C). These data suggest that the phenotypes in *shc-1(ok198);Is[daf-16::gfp]* animals require active DAF-2.

AKT-1 is known to act downstream of DAF-2 to inhibit DAF-16. Here, however, we observed that loss of *akt-1* and loss of *daf-2* have divergent consequences for DAF-16. To address whether the *daf-2* mutation could still suppress the defect in the basement membrane in *akt-1;shc-1* animals, we examined *shc-1(ok198);daf-2(e1370);akt-1(ok525)* triple mutant at 15°C, since only at this temperature the germline of the animals could proliferate after L3 stage. *daf-2* mutation suppressed gonad disruption in *shc-1(ok198);akt-1(ok525)* animals completely (Figure 4D, Figure S9), indicating that active IIR DAF-2 contributes to gonad disruption caused by DAF-16.

### AKT-2 and SGK-1 antagonize AKT-1 to affect gonadal integrity

As we observed antagonistic role of DAF-2 and AKT-1 affecting gonadal integrity, we further explored the downstream effectors of DAF-2 to antagonize AKT-1. In parallel to AKT-1, AKT-2 and SGK-1 also transmit input from DAF-2 to inhibit DAF-16. We asked whether AKT-2 or SGK-1 counteract AKT-1 to control gonadal integrity. Both *akt-2* and *sgk-1* RNAi clone strongly suppressed disruption of the gonad in *shc-1(ok198);akt-1(ok525)* mutant (Figure 4E and Table 2), indicating that AKT-2 and SGK-1 act downstream of DAF-2 to antagonize AKT-1.

### SGK-1 promotes tumorous germline proliferation in *shc-1;Is[daf-16::gfp]* animals

Next we asked whether DAF-2, AKT-1/2 or SGK-1 also promote tumorous germline proliferation in *shc-1;Is[daf-16::gfp]* animals. We quantified number of the germ cells of *shc-1;Is[daf-16::gfp]* L3 larvae in *daf-2*, *akt-1/akt-2* or *sgk-1* mutant background. Knock-down of *sgk-1* significantly reduced number of the germ cells (Figure 4F and Figure S10), suggesting SGK-1 contributes to proliferation of the germline tumor in *shc-1;Is[daf-16::gfp]* animals. Due to somatic missexpression of PGL-1 in *daf-2* and *akt-1/akt-2* animals [35], we were not able to quantify germ cells in these mutants (data not shown).

### Both active PI3K signaling and inactive JNK signaling contribute to the DAF-16–dependent defect in the gonadal basement membrane

**Figure 5:**
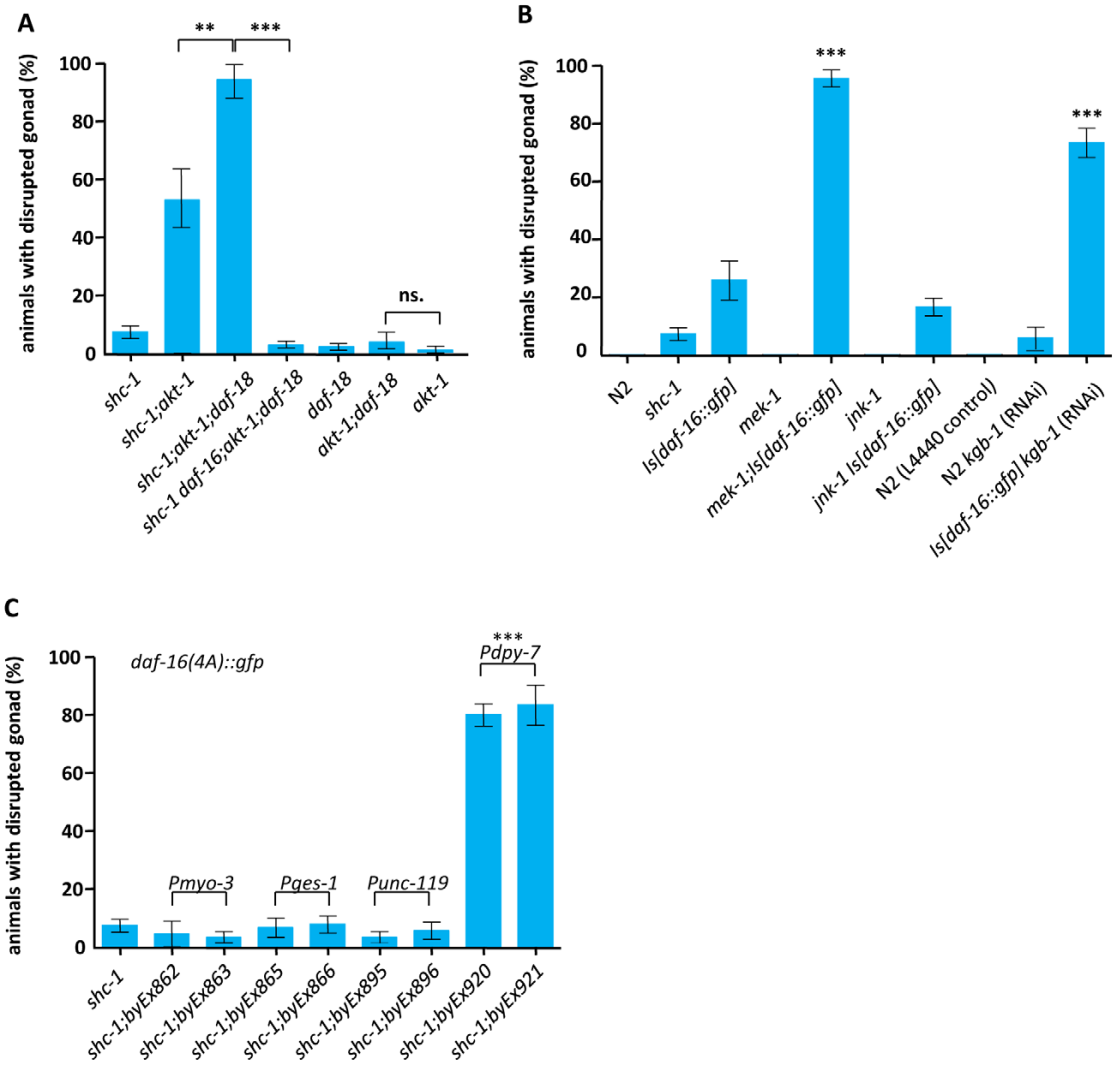
JNK signaling antagonizes hypodermal DAF-16 to ensure gonadal integrity. (A) *daf-18* mutation enhances disruption of the gonad in *shc-1(ok198);akt-1(ok525)* but not in *akt-1(ok525)* one day adult animals. (B) Knock-down of *mek-1* and *kgb-1* enhances the defect in *Is[daf-16::gfp]* animals. The mean values and statistic analysis are summarized in Table 2. (C) Expression of hypodermal *daf-16(4A)::gfp* leads to disruption of the gonadal basement membrane. *byEx* represents extrachromosomal transgene alleles which are summarized in Table 3. Mean values and statistic analysis of defect in gonadal integrity are summarized in Table 3.

SHC-1 has been shown to inhibit IIS to activate the downstream PI3K signaling [26]. Therefore, one may speculate that active DAF-16 in the presence of hyperactive PI3K signaling is the cause for gonad disruption. The PTEN homolog DAF-18 antagonizes PI3 kinase AGE-1. Therefore *daf-18* mutant should have enhanced PI3K signaling. To test whether active PI3K signaling contributes to the DAF-16 dependent phenotype, we analyzed *daf-18(e1375);akt-1(ok525)* and *shc-1(ok198);daf-18(e1375);akt-1(ok525)* animals. *daf-18* mutation did not enhance gonad disruption of *akt-1(ok525)* one day adult animals (Figure 5A, Table 2), suggesting that active PI3K is not sufficient for DAF-16 to degenerate the basement membrane. However, *daf-18* enhanced the defects in *shc-1(ok198);akt-1(ok525)* animals up to almost 100% and *daf-16* mutation completely suppressed the defect in *shc-1(ok198);daf-18(e1375);akt-1(ok525)* animals (Figure 5A and Table 2), indicating that active IIR/PI3K signaling assists DAF-16 to degenerate the gonadal basement membrane. Taking together, DAF-2 mediated PI3K signaling is necessary but not sufficient for DAF-16 to cause disruption of the gonad.

Besides negatively modulating DAF-2, SHC-1 also activates MEK-1/JNK-1 to affect DAF-16 [26]. Upon heavy metal stress response, SHC-1 also mediates the activation of MEK-1, which in turn phosphorylates and activates another JNK homolog, KGB-1 [27]. Therefore, in the *shc-1* mutant, hyperactive IIS is accomplished by inactive JNK signaling. Since hyperactive IIS is necessary but not sufficient for DAF-16 to derogate the gonadal basement membrane, we asked whether inactivated JNK signaling also plays a role. We generated *mek-1(ks54);Is[daf-16::gfp], jnk-1(gk7) Is[daf-16::gfp]* and *kgb-1(um3) Is[daf-16::gfp]* animals. Inactivation of JNK-1 did not further enhance disruption of the gonadal basement membrane in *Is[daf-16::gfp]* animals (Figure 5B, Table 2). 85% of *mek-1(ks54);Is[daf-16::gfp]* animals died at early larval stages. The remaining animals could develop to adulthood. However, 95.6% of these animals displayed germ cells outside of the gonad (Figure 5B and Table 2), phenocopying the *shc-1;Is[daf-16::gfp]* phenotype. Even though *mek-1;Is[daf-16::gfp]* animals showed more severe defects in early larval development than *shc-1(ok198);Is[[daf-16::gfp]* animals, fewer animals were sterile (29% *vs.* 43%; Table S2) and the fertile adult animals displayed a higher number of progeny (120 *vs.* 49; Table S2), suggesting that the early larval lethality is not due to a more severe defect in the basement membrane. All *kgb-1(um3) Is[daf-16::gfp]* animals died at larval stage. However, most of *Is[daf-16::gfp*] animals fed with *kgb-1* RNAi could bypass the larval lethality and in 75% of these one day old adult animals germ cells in the body cavity were observed (Figure 5B and Table 2). These results suggest that the antagonistic function of SHC-1 to DAF-16 is mediated via MEK-1 and KGB-1.

### Hypodermal expression of DAF-16 is sufficient to affect gonad integrity

The promoter used for the expression of *daf-16* in TJ356 *Is[daf-16::gfp]* is a 6 kb genomic region upstream of the exon 1 of *daf-16a* isoform, which triggers expression in the hypodermis, intestine, neurons and body wall muscle cells [29], [36]. Due to a general germline silencing of transgenic promoters in *C. elegans*, this *daf-16::gfp* transgene is probably not expressed in the germline. Endogenous DAF-16 in the germline or somatic gonad is not required for this phenotype, since no phenotypic difference of *shc-1;Is[daf-16::gfp]* animals was observed in *daf-16(+)* and *daf-16(mu86)* null mutant backgrounds (Table 2). We conclude that DAF-16 outside of the reproductive system is the most likely cause of the phenotype we describe here.

In order to test how DAF-16 in different tissues promotes the phenotype and rule out an involvement of weak expression of the *daf-16* transgene in the germline, we expressed the transgene in the specific somatic tissues. Since expressing the constitutively nuclear *daf-16(4A)::gfp* resulted in a stronger phenotype compared to wild type *daf-16::gfp*, we used *daf-16(4A)* for this analysis. Transgenic expression of *daf-16(4A)::gfp* in the neurons, intestine, or musculature did not increase the weak *shc-1(−)* phenotype (Figure 5C and Table 3). In contrast, expression of *daf-16(4A)::gfp* in the hypodermis was sufficient to cause disruption of the gonad in 80% of one day adult *shc-1(−)* animals, suggesting that hypodermal DAF-16 causes disruption of the gonadal basement membrane cell-nonautonomously.

## Discussion

### Two opposing qualities of DAF-16 signaling affect lifespan differently

In this manuscript we for the first time describe two qualities of DAF-16 function that affect longevity in opposite way. In contrast to the known lifespan extending effect, we discover that DAF-16 can shorten lifespan by inducing a tumor-like germline phenotype. It has probably affected the outcome of previously described experiments, however, to our knowledge this has not been reported before [17]–[36].

The starting point of our experiments was that we, as others before, failed to increase the lifespan of wild type (*daf-2* +/+) animals by expressing transgenic copies of *daf-16*, unless the lifespan assays were performed in the presence of FUDR [37] (Figure 1B). In this respect *daf-16* differs from other key regulators of stress response and lifespan such as *skn-1*, which typically shorten lifespan when inactivated, and increase lifespan when being overexpressed as a transgene. Although *daf-16* transgene rescued the short life-span of *daf-2;daf-16* double mutants, it did not or only modestly increases lifespan in wild type background [17]–[36]. It has been suggested that in a *daf-2(+)* background, AKT phosphorylation results in cytoplasmic retention of these extra copies of DAF-16, rendering them inactive [36]. However, phosphorylation was not sufficient to prevent the ability of transgenic SKN-1 to extend lifespan [38]. We found here that the lifespan extending effect of DAF-16 was balanced by its lifespan shortening effect which induced tumor-like growth in the germline. Consistently, blocking germline proliferation by using the cytostatic FUDR or a mutation inhibiting Notch signaling in the germline was sufficient to prevent negative aspects of DAF-16 signaling and increased lifespan of wild type animals containing a *daf-16* transgene (Figure 1B and 1C).

### DAF-16–mediated signaling results in a tumor-like germline phenotype

We found that not only a *daf-16* transgene, but also a combination of mutants in the IIS pathway affecting DAF-16 activity caused a pleiotropic phenotype in the reproductive system. This phenotype occurred at low penetrance in wild type animals carrying the *daf-16* transgene, which may be the reason why it had escaped detection in previous studies. The penetrance of this phenotype was strongly enhanced in *shc-1* mutant background. Its most prominent phenotypic aspect was the disruption of the gonadal basement membrane at the proximal gonad adjacent to the developing somatic gonad primordium, so that eventually germline cells leaked into the body. We counted about 30% more germ cells in disrupted gonad arms of *shc-1;Is[daf-16::gfp]* L3 larvae compared to wild type animals (Figure 2K). A co-segregation of both phenotypic aspects was particularly obvious in animals in which only one gonad arm was disrupted, and typically contained significantly more germ cells than the other intact gonad arm. This phenotype is distinct from that of *gld-1* mutants, in which the basement membrane ruptures in adult animals due to a massive excess of germ cells. *gld-1* mutants at the equivalent L3 stage still have intact gonad arms despite their increased number of germ cells [39]. This indicates that in *gld-1* mutants, but not in animals described in this study, the basement membrane of L3 animals is mechanically strong enough to withstand an increase in germline nuclei. It is therefore possible that the basement membrane of the gonad in *shc-1;Is[daf-16]* has a defect on its own, similar as e.g. seen in *ten-1*, *dgn-1*, *ina-1* and *epi-1* mutants, which also show disruption of gonadal basement membrane, but without an increase in germline proliferation [40]. An interesting hypothesis is that that the abnormally proliferating germ cells invade into the surrounding extracellular matrix. Although we observed germ cells that penetrate the basement membrane, and dissection and extraction of an intact gonad in those animals failed due to their leakage, we cannot prove at this point that this is an active process. Nevertheless, we discover for the first time that mutations in IIS result in both germline hyperplasia and disruption of the gonadal basement membrane in *C. elegans*, a phenotype that opposes the beneficial effects of DAF-16 on increasing healthspan.

### DAF-16 activation in the hypodermis is sufficient to signal to the germline

The phenotype in the reproductive system was also observed in *shc-1* mutants carrying extrachromosomal arrays with *daf-16*. Such arrays in *C. elegans* are typically not expressed in the germline [41]. In agreement with this generally accepted notion, we never observed germline expression of the GFP tagged *daf-16* transgenes. One possibility is that overexpression of DAF-16 in the somatic tissues leads to activation of germline DAF-16, since it has been shown that active DAF-16 in one tissue elevated its activity in other tissues [42]. However, we observed no difference between *shc-1;Is[daf-16::gfp]* and *shc-1 daf-16;Is[daf-16::gfp]* animals, the latter lacking endogenous *daf-16* in the germline [19] (Table 2). Therefore, we consider it more likely that *daf-16* expression outside the reproductive system is the cause of the observed phenotype. The fact that expressing *daf-16* in the hypodermis was sufficient to provoke this phenotype (Figure 5C) further excluded an involvement of very low levels of transgenic DAF-16 in the germline. These data suggest that DAF-16 mediates a cell-nonautonomous signaling to affect germline proliferation and basement membrane integrity.

### SHC-1 regulates both IIS and MAPK signaling pathway to antagonize DAF-16 influence on the germline

Mutation in *shc-1* strongly enhanced the germline and gonad phenotype of *Is[daf-16::gfp]* animals. In addition, we observed that DAF-16(4A) was nuclearly localized in both *shc-1(+)* and *shc-1(−)* animals (Figure 3D), while disruption of the gonad was only detectable in *shc-1(−)* background (Figure 3C). These data favor a model that SHC-1, in addition to its role in affecting the subcellular localization of DAF-16 upon stress response, can also antagonize nuclear DAF-16. Notably, SHC-1 is localized in both cytoplasm and nucleus [26]. Our data indicate that both active IIS and inactive JNK signaling contribute to the DAF-16 dependent phenotype in the reproductive system (Figure 4B, 4D, 5A and 5B). This may also explain why the *mek-1;Is[daf-16::gfp]* strain displayed a less severe basement membrane phenotype than *shc-1;Is[daf-16::gfp]* animals, as only SHC-1 affects both IIS and JNK signaling [26]. Our data also explain previous, contradictory data concerning the lifespan of *mek-1* mutants. We reported previously that *mek-1* mutants were short lived, while Oh and colleagues found *mek-1* mutants to have a lifespan indistinguishable from wild type [9], [26]. We found that both *mek-1* and *shc-1* mutant animals lived as long as wild type worms in the presence of the drug FUDR that blocked germline proliferation (Table 2). However, in the absence of FUDR, *mek-1* animals suffered from a pronounced reduction of lifespan that we can now attribute to defects in the reproductive system, rather than to the sensitivity for reactive oxygen species stress as proposed before [26].

### AKT-2 and SGK-1 counteract AKT-1 to control DAF-16 signaling to the germline

We found that in a *daf-2* loss-of-function mutant the DAF-16 mediated gonad dysintegrity phenotype of both *shc-1;Is[daf-16::gfp]* and *shc-1;akt-1* animals was reduced (Figure 4B and 4D), thus abrogating lethality in early adulthood (Figure 4C). This was a surprising result, since our experiments so far had indicated that this gonad phenotype was a consequence of too much DAF-16 activity, and the *daf-2* mutation should further increase DAF-16 activity. The PTEN homolog DAF-18 antagonizes the DAF-2 and PI3 kinase/AGE-1 pathway, thus a loss-of-function mutant is supposed to result in reduced DAF-16 activity via increased IIS. However, the *daf-18* loss-of-function mutation further enhanced the phenotype of a *shc-1;akt-1* mutant (Figure 5A). In summary, mutations in both IIS pathway genes *daf-2* and *daf-18* behave opposite to their known roles in the canonical IIS pathway. Similarly, while the increase of the phenotype in an *akt-1* loss-of-function mutant correlated well with the increase of DAF-16 activity, *akt-2* and *sgk-1* mutations opposed *akt-1*, and behaved like *daf-2* described above. We conclude that, while *daf-2*, *akt-1*, *akt-2*, and *sgk-1* loss-of-function mutants all result in a longevity and stress resistance phenotype consistent with upregulation of DAF-16 nuclear activity, they differ in the way they regulate DAF-16 activity to cause the gonad phenotype.

One possible explanation for the antagonistic effect of distinct IIs pathway mutants is that *daf-2*, *akt-2*, and *sgk-1* may provoke a distinct phosphorylation pattern of DAF-16 compared to *akt-1*, resulting in different outputs of DAF-16 transcriptional targets that affect germline proliferation/gonadal integrity compared to longevity and stress resistance. Whereas the *akt-1(ok52*5*)* mutation might only prevent AKT-1 mediated DAF-16 phosphorylation, *daf-2(e1370)* supposedly reduces both AKT-1, AKT-2, and SGK-1 phosphorylation, and this may result in controlling distinct downstream genes for long lifespan and gonad integrity, respectively. We consider this model less likely, since there is currently no evidence for C. elegans AKT-1 phosphorylating distinct sites in DAF-16 compared to SGK-1 or AKT-2 [7]. However, we cannot exclude this possibility based on the existing data.

Another possible scenario is that mutation in *daf-2*, *akt-2*, and *sgk-1* mediated activation of DAF-16 affects different tissues than *akt-1* knock-down mediated activation of DAF-16, suggesting that the germline reads out and responds to a balance of DAF-16 activities in somatic tissues. This way, DAF-2 could prevent DAF-16 from counteracting hypodermal DAF-16 in some tissues, e.g. the intestine. It has been previously shown that DAF-2 promotes larval germline proliferation via inactivating germline DAF-16 [19]. Together with our results this suggests that DAF-16 activities in the hypodermis and the germline may antagonize each other. Moreover, previous studies suggested that AKT-1, AKT-2 and SGK-1 have different tissue specificity, expression levels, patterns, or activities to control DAF-16 [4], [7]. Notably, AKT-1 is expressed in the hypodermis, whereas AKT-2 and SGK-1 are probably not. Consistently, neither down-regulation of *akt-2* nor *sgk-1* caused disruption of the gonad in *shc-1* mutant background (Figure 4A), but instead were both able to suppress the phenotype of *akt-1;shc-1* mutants (Figure 4E). Based on our results presented here, we suggest that AKT-1 is active in the hypodermis to prevent DAF-16 mediated signal to the reproductive system, whereas AKT-2, SGK-1 and even AKT-1 in other tissues may inhibit DAF-16 activity that counteracts hypodermal DAF-16. Such antagonistic roles of SGK-1 and AKT-1 have been reported in regulation of lifespan [43]. Additional tissue specific studies of these three AGC family kinases will enable a further understanding of such interactions across tissue boundaries.

### FOXO and tumor formation

It has been shown in different organisms that FOXO/DAF-16 functions as a tumor suppressor in a variety of cancers by promoting apoptosis or cell cycle arrest [44]. However, several recent data indicate that the function of FOXO/DAF-16 may be more complex than previously thought. An essential role of FOXO3a was proposed in the maintenance of cancer stem cells that are responsible for the reoccurrence of chronic myeloid leukemia [15]. In acute myeloid leukemia FOXO1/3/4 promote leukemic growth and maintenance by inhibiting myeloid maturation and apoptosis [45]. In *C elegans*, some DAF-16 target genes were identified which stimulate *gld-1* germline tumor [46]. All these studies indicate that the function of FOXO/DAF-16 proteins is highly dependent on the cellular context. In mouse, FOXO3a promotes tumor cell invasion through the induction of matrix metalloproteases [47]. Our data show that FOXO/DAF-16 can also promote germline hyperplasia and disruption of the surrounding extracellular matrix through cell-nonautonomous signaling. It will be of great interest to further identify the signaling molecules to the germline activated by DAF-16, and to investigate whether the gonad dysintegrity phenotype we described involves active invasions of the germ cells into the extracellular matrix and, therefore, resembles the behavior of metastatic tumor cells.

## Materials and Methods

Information about strains and constructs used are described in Text S1.

### Transgenic strains

To generate transgenic animals carrying wild type *daf-16::gfp* or *daf-16(4A)::gfp*, the corresponding constructs were injected into wild type or *shc-1(ok198)* animals (for *byEx* numbers, see Table 3). 20 ng/µl pRF4 *rol-6* (*su1006*) was used as co-injection marker. GFP expression of the transgenic animals was confirmed using fluorescent microscopy.

### Antibody staining

A formaldehyde fixation procedure was used (Text S1) for the whole worm staining with 1∶200 anti-PGL-1 antibody (a gift from Dr. Susan Strome). Staining of dissected gonads was not possible because of the disruption of the gonad.

### MitoTracker staining of the basement membrane

Basement membranes were stained with MitoTracker Red CMXRos (invitrogen) by placing worms in a solution of 10 µM MitoTracker Red at 25°C for 2 hours. The worms were then allowed to recover for 30 minutes on an NGM agar plate and analyzed.

### Lifespan assay

Lifespan assays were initiated at the L4 larval stage. Synchronized animals were raised at 15°C prior to lifespan analysis. Then, L4 animals were transferred to the respective temperatures for the assays and examined every day. Animals that showed no response to touch were scored as dead. In assays without FUDR treatment animals were transferred every day onto new plates during the reproductive period. Animals died because of bagging of larvae were censored. In the lifespan assay with FUDR treatment, animals were transferred onto the agar plates containing 0.1 mg/ml FUDR and *E.coli* 24 hours after the L4 larval stage. These were raised on the FUDR containing plates for four days and then transferred onto new plates without FUDR. FUDR was administrated to adult instead of L4 animals in order to exclude a possible interference from seam cell development to the reproductive system, since the epidermal seam cells undergo a final mitotic division at the L4 to adult molt and DAF-16 mediated signaling is sent from the hypodermis. All the lifespan assays were performed at 20°C with exception of those with *glp-1(q231)* and *daf-2(e1370)* mutants, as *glp-1(q231)* required 25°C to inactivate GLP-1 and *daf-2(e1370);shc-1(ok198);Is[daf-16::gfp]* could develop to adulthood only at 15°C. All of the lifespan assays were performed at least twice. The short lifespan of *shc-1(ok198);Is[daf-16::gfp]* can only be observed, when no censoring for adult animals dying due to the development defect is done. As reported previously by us (Neumann-Haefelin et al., 2008), *Is[daf-16::gfp]* partially extends lifespan of *shc-1(ok198)* mutants, when adult animals dying due to the development defect are censored.

### Scoring of gonad disruption

All animals in the tests except those with *daf-2(e1370);shc-1(ok198);Is[daf-16::gfp]* and *daf-2(e1370);shc-1(ok198);akt-1(ok525)*, were raised at 20°C and analyzed via DIC microscopy 24 hours after L4 stage. Animals in which at least one germ cell was detected outside of the gonad were scored as positive. The percentage of positive one day old animals was calculated. Per test thirty animals were examined and each test was performed at least three times. *shc-1(ok198);daf-2(e1370);Is[daf-16::gfp]* and *shc-1(ok198);daf-2(e1370);akt-1(ok525)* animals were raised at 15°C and analyzed 24 hours after L4 larval stage.

### Quantification of the germ cells

The numbers of anti-PGL-1 positive cells were quantified at the time point that vulva induction takes place.

### Statistic analysis

GraphPad Prism 4.0 software (GraphPad Software Inc., San Diego, USA) was used to calculate mean value and to perform statistical analysis.

## Supporting Information

Figure S1 ***glp-1(q231)*** **three day old adult animals display atrophy of the intestine and accumulation of undigested** ***E. coli*** **in the intestinal lumen.** The arrow heads point to the intestinal cells in long-lived *glp-1(e2141)* animals. The arrows point to intestinal atrophy in *glp-1(q231)* animals. Animals are raised at 25°C from L2 larval stage to inactivate GLP-1.
(DOCX)

Figure S2 **Knock-down of** ***glp-1*** **extend lifespan of** ***shc-1(ok198);Is[daf-16::gfp]*** **animals at 20°C.** Mean lifespan: N2: 17.6 days (n = 82); *glp-1(q231)*: 16.8 days (n = 107); *shc-1(ok198);Is[daf-16::gfp]*: 6.4 days (n = 86); *shc-1(ok198);glp-1(q231);Is[daf-16::gfp]*: 22.7 days (n = 135). Animals were shifted to 25°C at the L2 larval stage to cease germline proliferation and shifted back to 20°C 24 hours after L4 stage. Adult lifespan was performed at 20°C. This Figure is related to the main Figure 1.
(DOCX)

Figure S3 **Inactivation of GLP-1 suppresses tumorous germline phenotype in *shc-1;Is[daf-16::gfp]* animals.** A: *shc-1(ok198);Is[daf-16::gfp]* one day adult; B: *shc-1(ok198) glp-1(q231);Is[daf-16::gfp]* one day adult; C: *shc-1(ok198) glp-1(e2141) ;Is[daf-16::gfp]* one day adult. Animals were raised at 25°C from L2 larval stage. In both *glp-1* alleles there were only somatic gonad and some sperms observed. This Figure is related to the main Figure 2.
(DOCX)

Figure S4 **DTC migration defect in** ***shc-1;Is[daf-16::gfp]*** **animals.**
(DOCX)

Figure S5 **Staining of the gonadal basement membrane in** ***shc-1;Is[daf-16::gfp]*** **L3 animals.** The white arrows point to the somatic gonad primordium. The white arrow heads donate the germ cells leaking out of the gonad.
(DOCX)

Figure S6 **Residue expression of** ***daf-16::gfp*** **in** ***shc-1;Is[daf-16::gfp]*** **animals upon** ***daf-16*** **RNAi knock-down.** A: *shc-1;Is[daf-16::gfp]* on L4440 control; B: *shc-1;Is[daf-16::gfp]* on *daf-16* RNAi, the arrows point to the residue GFP expression; C: quantification of fluorescent: *shc-1(ok198)* as background control: 9.67±3.58 Grey (n = 31), *shc-1;Is[daf-16::gfp]*(L4440): 1876.02±231.78 Grey (n = 31), *shc-1 daf-16(RNAi);Is[daf-16::gfp]*: 90.62±40.15 Grey (n = 28), P<0.001 compared to *shc-1;Is[daf-16::gfp]*(L4440).
(DOCX)

Figure S7 **GFP intensity in** ***shc-1(ok198)*** **animals carrying** ***daf-16*** **transgenes.** The Figure A and Figure B (*shc-1;Ex[daf-16::gfp]/byEx879*) have the same exposure time. The exposure time was 42 ms for Figure C and 5,000 ms for Figure D, respectively. *shc-1(ok198);Ex[daf-16::gfp]* and *shc-1(ok198);Ex[daf-16(4A)::gfp]* animals display comparable phenotype in the basement membrane (45.5% vs. 57.2% of one day adults display disrupted gonad). Scale bar 10 µm. This Figure is related to the main Figure 3.
(DOCX)

Figure S8 **DIC images of wild type and** ***shc-1(ok198);daf-2(e1370);Is[daf-16::gfp]*** **animals grown at 20°C.** DIC images of wild type and *shc-1(ok198);daf-2(e1370);Is[daf-16::gfp]* animals were taken three days and six days after hatching, respectively. The gonad of wild type animals contained germ cells in mitotic (mito), meiotic (meiot) stages, oocytes (o), sperm (sp) in the spematheca and embryos (em) in the uterus. The gonad of *shc-1(ok198);daf-2(e1370);Is[daf-16::gfp]* animals contained less than 50 germ cells whereas vulva (v) induction still occurred. No oocyte or sperm could be identified in the gonad of *shc-1(ok198);daf-2(e1370);Is[daf-16::gfp]* animals. Scale bar 10 μm. This figure is related to the main Figure 4.
(DOCX)

Figure S9 **DIC images of *shc-1(ok198);akt-1(ok525)* and *shc-1(ok198);akt-1(ok525);daf-2(e1370)* animals.** The arrow and arrowheads point to the ruptured gonad and germ cells outside the gonad, respectively. This figure is related to the main Figure 4.
(DOCX)

Figure S10 **anti-PGL-1 antibody staining (I) of** ***shc-1(ok198);Is[daf-16::gfp]*** **and** ***shc-1(ok198);sgk-1(ok538);Is[daf-16::gfp]*** **L3 larvae.** In *shc-1(ok198);Is[daf-16::gfp]* animals the anterior gonad is disrupted and contain more germ cells than the posterior intact one or that in *shc-1(ok198);sgk-1(ok538);Is[daf-16::gfp]* animals. This Figure is related to the main Figure 4.
(DOCX)

Movie S1 **Z-stack image of PGL-1 antibody staining of** ***shc-1(ok198);Is[daf-16::gfp]*** **L3 animals.**
(MOV)

Table S1 **Lifespan with** ***glp-1(q231).***
(DOCX)

Table S2 **Brood size and sterility.**
(DOCX)

Text S1 **Supplemental experimental procedure.**
(DOCX)
