# Supplementary material for: Cell-Nonautonomous Signaling of FOXO/DAF-16 to the Stem Cells of Caenorhabditis elegans
Source: PLoS Genet. 2012 Aug 16;8(8):e1002836. doi: 10.1371/journal.pgen.1002836 (PMC3420913; doi:10.1371/journal.pgen.1002836)
Supplement: Figure S1 — glp-1(q231) three day old adult animals display atrophy of the intestine and accumulation of undigested E. coli in the intestinal lumen. The arrow heads point to the intestinal cells in long-lived glp-1(e2141) animals. The arrows point to intestinal atrophy in glp-1(q231) animals. Animals are raised at 25°C from L2 larval stage to inactivate GLP-1. (DOCX) [file pgen.1002836.s001.docx]

**S1**

**Figure S1** *glp-1(q231)* three day old adult animals display atrophy of the intestine and accumulation of undigested *E. coli* in the intestinal lumen.
