# Supplementary material for: Cell-Nonautonomous Signaling of FOXO/DAF-16 to the Stem Cells of Caenorhabditis elegans
Source: PLoS Genet. 2012 Aug 16;8(8):e1002836. doi: 10.1371/journal.pgen.1002836 (PMC3420913; doi:10.1371/journal.pgen.1002836)
Supplement: Figure S2 — Knock-down of glp-1 extend lifespan of shc-1(ok198);Is[daf-16::gfp] animals at 20°C. Mean lifespan: N2: 17.6 days (n = 82); glp-1(q231): 16.8 days (n = 107); shc-1(ok198);Is[daf-16::gfp]: 6.4 days (n = 86); shc-1(ok198);glp-1(q231);Is[daf-16::gfp]: 22.7 days (n = 135). Animals were shifted to 25°C at the L2 larval stage to cease germline proliferation and shifted back to 20°C 24 hours after L4 stage. Adult lifespan was performed at 20°C. This Figure is related to the main Figure 1. (DOCX) [file pgen.1002836.s002.docx]

**S2**

**Figure S2.** Knock-down of *glp-1* extend lifespan of *shc-1(ok198);Is[daf-16::gfp]* animals at 20°C.
