# Supplementary material for: Cell-Nonautonomous Signaling of FOXO/DAF-16 to the Stem Cells of Caenorhabditis elegans
Source: PLoS Genet. 2012 Aug 16;8(8):e1002836. doi: 10.1371/journal.pgen.1002836 (PMC3420913; doi:10.1371/journal.pgen.1002836)
Supplement: Figure S3 — Inactivation of GLP-1 suppresses tumorous germline phenotype in shc-1;Is[daf-16::gfp] animals. A: shc-1(ok198);Is[daf-16::gfp] one day adult; B: shc-1(ok198) glp-1(q231);Is[daf-16::gfp] one day adult; C: shc-1(ok198) glp-1(e2141) ;Is[daf-16::gfp] one day adult. Animals were raised at 25°C from L2 larval stage. In both glp-1 alleles there were only somatic gonad and some sperms observed. This Figure is related to the main Figure 2. (DOCX) [file pgen.1002836.s003.docx]

**S3**

**Figure S3.** Inactivation of GLP-1 suppresses tumorous germline phenotype in *shc‑1;Is[daf‑16::gfp]* animals.
