# Supplementary material for: Cell-Nonautonomous Signaling of FOXO/DAF-16 to the Stem Cells of Caenorhabditis elegans
Source: PLoS Genet. 2012 Aug 16;8(8):e1002836. doi: 10.1371/journal.pgen.1002836 (PMC3420913; doi:10.1371/journal.pgen.1002836)
Supplement: Figure S4 — DTC migration defect in shc-1;Is[daf-16::gfp] animals. (DOCX) [file pgen.1002836.s004.docx]

**S4**

**Figure S4**. DTC migration defect in *shc-1;Is[daf-16::gfp]* animals.
