# Supplementary material for: Cell-Nonautonomous Signaling of FOXO/DAF-16 to the Stem Cells of Caenorhabditis elegans
Source: PLoS Genet. 2012 Aug 16;8(8):e1002836. doi: 10.1371/journal.pgen.1002836 (PMC3420913; doi:10.1371/journal.pgen.1002836)
Supplement: Figure S5 — Staining of the gonadal basement membrane in shc-1;Is[daf-16::gfp] L3 animals. The white arrows point to the somatic gonad primordium. The white arrow heads donate the germ cells leaking out of the gonad. (DOCX) [file pgen.1002836.s005.docx]

**S5**

**Figure S5**. Staining of the gonadal basement membrane in *shc-1;Is[daf-16::gfp]* L3 animals.
