# Supplementary material for: Cell-Nonautonomous Signaling of FOXO/DAF-16 to the Stem Cells of Caenorhabditis elegans
Source: PLoS Genet. 2012 Aug 16;8(8):e1002836. doi: 10.1371/journal.pgen.1002836 (PMC3420913; doi:10.1371/journal.pgen.1002836)
Supplement: Figure S6 — Residue expression of daf-16::gfp in shc-1;Is[daf-16::gfp] animals upon daf-16 RNAi knock-down. A: shc-1;Is[daf-16::gfp] on L4440 control; B: shc-1;Is[daf-16::gfp] on daf-16 RNAi, the arrows point to the residue GFP expression; C: quantification of fluorescent: shc-1(ok198) as background control: 9.67±3.58 Grey (n = 31), shc-1;Is[daf-16::gfp](L4440): 1876.02±231.78 Grey (n = 31), shc-1 daf-16(RNAi);Is[daf-16::gfp]: 90.62±40.15 Grey (n = 28), P<0.001 compared to shc-1;Is[daf-16::gfp](L4440). (DOCX) [file pgen.1002836.s006.docx]

**S6**

**Figure S6**. Residue expression of *daf-16::gfp* in *shc-1;Is[daf-16::gfp]* animals upon *daf-16* RNAi knock-down.
