# Supplementary material for: Cell-Nonautonomous Signaling of FOXO/DAF-16 to the Stem Cells of Caenorhabditis elegans
Source: PLoS Genet. 2012 Aug 16;8(8):e1002836. doi: 10.1371/journal.pgen.1002836 (PMC3420913; doi:10.1371/journal.pgen.1002836)
Supplement: Figure S7 — GFP intensity in shc-1(ok198) animals carrying daf-16 transgenes. The Figure A and Figure B (shc-1;Ex[daf-16::gfp]/byEx879) have the same exposure time. The exposure time was 42 ms for Figure C and 5,000 ms for Figure D, respectively. shc-1(ok198);Ex[daf-16::gfp] and shc-1(ok198);Ex[daf-16(4A)::gfp] animals display comparable phenotype in the basement membrane (45.5% vs. 57.2% of one day adults display disrupted gonad). Scale bar 10 µm. This Figure is related to the main Figure 3. (DOCX) [file pgen.1002836.s007.docx]

**S7**

**Figure S7.** GFP intensity in *shc‑1(ok198)* animals carrying *daf-16* transgenes.
