# Supplementary material for: Cell-Nonautonomous Signaling of FOXO/DAF-16 to the Stem Cells of Caenorhabditis elegans
Source: PLoS Genet. 2012 Aug 16;8(8):e1002836. doi: 10.1371/journal.pgen.1002836 (PMC3420913; doi:10.1371/journal.pgen.1002836)
Supplement: Figure S8 — DIC images of wild type and shc-1(ok198);daf-2(e1370);Is[daf-16::gfp] animals grown at 20°C. DIC images of wild type and shc-1(ok198);daf-2(e1370);Is[daf-16::gfp] animals were taken three days and six days after hatching, respectively. The gonad of wild type animals contained germ cells in mitotic (mito), meiotic (meiot) stages, oocytes (o), sperm (sp) in the spematheca and embryos (em) in the uterus. The gonad of shc-1(ok198);daf-2(e1370);Is[daf-16::gfp] animals contained less than 50 germ cells whereas vulva (v) induction still occurred. No oocyte or sperm could be identified in the gonad of shc-1(ok198);daf-2(e1370);Is[daf-16::gfp] animals. Scale bar 10 μm. This figure is related to the main Figure 4. (DOCX) [file pgen.1002836.s008.docx]

**S8**

**Figure S8.** DIC images of wild type and *shc‑1(ok198);daf‑2(e1370);Is[daf‑16::gfp]* animals grown at 20°C.
