# Supplementary figures and images for: Cell-Nonautonomous Signaling of FOXO/DAF-16 to the Stem Cells of Caenorhabditis elegans
Source: PLoS Genet. 2012 Aug 16;8(8):e1002836. doi: 10.1371/journal.pgen.1002836 (PMC3420913; doi:10.1371/journal.pgen.1002836)

**S9**

**
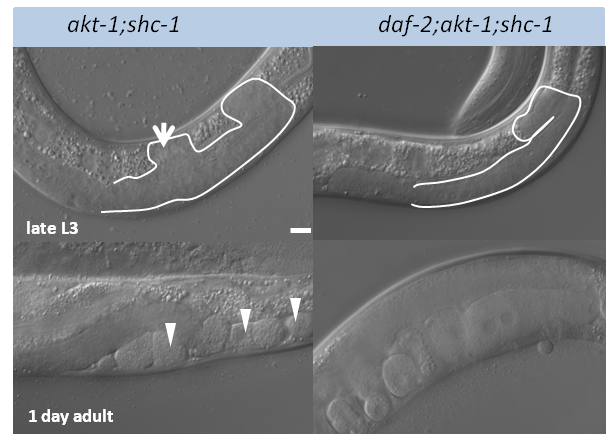
**

**Figure S9.** DIC images of *shc-1(ok198);akt‑1(ok525)* and *shc‑1(ok198);akt‑1(ok525);daf‑2(e1370)* animals.

Supplement: Figure S9 — DIC images of shc-1(ok198);akt-1(ok525) and shc-1(ok198);akt-1(ok525);daf-2(e1370) animals. The arrow and arrowheads point to the ruptured gonad and germ cells outside the gonad, respectively. This figure is related to the main Figure 4. (DOCX) [file pgen.1002836.s009.docx]
