# Supplementary material for: Cell-Nonautonomous Signaling of FOXO/DAF-16 to the Stem Cells of Caenorhabditis elegans
Source: PLoS Genet. 2012 Aug 16;8(8):e1002836. doi: 10.1371/journal.pgen.1002836 (PMC3420913; doi:10.1371/journal.pgen.1002836)
Supplement: Figure S10 — anti-PGL-1 antibody staining (I) of shc-1(ok198);Is[daf-16::gfp] and shc-1(ok198);sgk-1(ok538);Is[daf-16::gfp] L3 larvae. In shc-1(ok198);Is[daf-16::gfp] animals the anterior gonad is disrupted and contain more germ cells than the posterior intact one or that in shc-1(ok198);sgk-1(ok538);Is[daf-16::gfp] animals. This Figure is related to the main Figure 4. (DOCX) [file pgen.1002836.s010.docx]

**S10**

**Figure S10.** Anti-PGL‑1 antibody staining (I) of *shc‑1(ok198);Is[daf‑16::gfp]* and *shc‑1(ok198);sgk‑1(ok538);Is[daf‑16::gfp]* L3 larvae.
