# Supplementary material for: Cell-Nonautonomous Signaling of FOXO/DAF-16 to the Stem Cells of Caenorhabditis elegans
Source: PLoS Genet. 2012 Aug 16;8(8):e1002836. doi: 10.1371/journal.pgen.1002836 (PMC3420913; doi:10.1371/journal.pgen.1002836)
Supplement: Table S1 — Lifespan with glp-1(q231). (DOCX) [file pgen.1002836.s012.docx]

**Table S1. Lifespan with *glp-1(q231)***

| **Time point of the temperature shift** | Stains | Mean lifespan  (days) | Numbers of examined animals | P Value | Change in mean lifespan |
| --- | --- | --- | --- | --- | --- |
| L2 | N2 | 9.6 | 127 |  |  |
|  | *glp-1(q231)* | 8.2 | 130 |  | 85.4%* |
|  | *shc-1(ok198);Is[daf-16::gfp*] | 5.0 | 139 |  |  |
|  | *glp-1;shc-1;Is[daf-16::gfp]* | 16.1 | 172 | <0.0001 | 340%** |
| L3 | N2 | 11.1 | 139 |  |  |
|  | *glp-1(q231)* | 9.8 | 213 |  | 88.3%* |
|  | *shc-1(ok198);Is[daf-16::gfp*] | 4.8 | 129 |  |  |
|  | *glp-1;shc-1;Is[daf-16::gfp]* | 11.9 | 110 | <0.0001 | 248.9%** |
| L4 | N2 | 10.2 | 92 |  |  |
|  | *glp-1(q231)* | 8.9 | 170 |  | 87.3%* |
|  | *shc-1(ok198);Is[daf-16::gfp*] | 5.0 | 144 |  |  |
|  | *glp-1;shc-1;Is[daf-16::gfp]* | 8.6 | 183 | <0.0001 | 172.0%** |

*: normalized to N2; **: normalized to *shc-1(ok198);Is[daf-16::gfp]*; P value relative to *shc‑1(ok198);Is[daf‑16::gfp]*

This table is related to the main Figure 1.
