# Supplementary material for: Cell-Nonautonomous Signaling of FOXO/DAF-16 to the Stem Cells of Caenorhabditis elegans
Source: PLoS Genet. 2012 Aug 16;8(8):e1002836. doi: 10.1371/journal.pgen.1002836 (PMC3420913; doi:10.1371/journal.pgen.1002836)
Supplement: Table S2 — Brood size and sterility. (DOCX) [file pgen.1002836.s013.docx]

**Table S2. Brood size and sterility**

| strains | mean brood size (n) | sterile (n) |
| --- | --- | --- |
| N2 | 348 (10) | 0% (348) |
| *shc-1(ok198)* | 322 (10) | 0% (461) |
| *shc-1(tm1729)* | 324 (5) | 0% (218) |
| *Is[daf-16::gfp]* | 154 (9) | 0% (329) |
| *shc-1(ok198);Is[daf-16::gfp*] | 49 (20) | 43% (194) |
| *shc-1(tm1729);Is[daf-16::gfp*] | 31 (10) | 56% (171) |
| *shc-1(ok198);Is[daf-16::gfp*]+*daf-16* RNAi | 262 (8) | 0% (175) |
| *mek-1(ks54);Is[daf-16::gfp]* | 120 (9) | 29% (152) |
| N2* | 291 (6) | 0% (349) |
| *daf-2(e1370)** | 276 (10) | 0% (120) |
| *shc-1(ok198);Is[daf-16::gfp*]* | 27 (16) | 50% (194) |
| *shc-1;daf-2;Is[daf-16::gfp]** | 99 (21) | 0% (110) |

*: animals maintained at 15°C; n: numbers of examined animals.
