# Supplementary material for: Cell-Nonautonomous Signaling of FOXO/DAF-16 to the Stem Cells of Caenorhabditis elegans
Source: PLoS Genet. 2012 Aug 16;8(8):e1002836. doi: 10.1371/journal.pgen.1002836 (PMC3420913; doi:10.1371/journal.pgen.1002836)
Supplement: Text S1 — Supplemental experimental procedure. (DOCX) [file pgen.1002836.s014.docx]

**Text S1**

**Strains**

The strains used were as follow: wild-type N2 Bristol, NH3119 *shc-1(ok198*) (newly outcrossed five times to N2), *shc-1(tm1729)* (newly outcrossed six times to N2), TJ356 *Is[daf-16::gfp]* (newly outcrossed five times to N2), BR5082 *shc‑1(ok198);Is[daf-16::gfp],* BR5106 *shc‑1(tm1729);Is[daf-16::gfp]*, JK509 *glp-1(q231)* (newly outcrossed four times to N2)*,* BR4880 *shc‑1(ok198);glp‑1(q231);Is[daf‑16::gfp]*; CF1038 *daf‑16(mu86),* BR4912 *daf-16(mu86) shc-1(ok198)*; BR5129 *shc-1(ok198) daf‑16(mu86);Is[daf-16::gfp],* GR1307 *daf-16(mgDF50)*, BR3572 *shc‑1(ok198) daf-16(mgDF50),* CB1370 *daf-2(e1370)* (newly outcrossed five times to N2), BR4773 *shc‑1(ok198);daf‑2(e1370);Is[daf‑16::gfp]*, RB759 *akt‑1(ok525)*, BR5964 *shc‑1(ok198);akt‑1(ok525),* BR5999 *shc‑1(ok198) daf‑16(mgDF50);akt‑1(ok525),* VC204 *akt-2(ok393)*, BR5965 *akt‑2(ok393);shc-1(ok198),* BR6235 *akt‑1(ok525);daf‑2(e1370);shc‑1(ok198),* CB1375 *daf‑18(e1375)*, BR6335 *daf‑18(e1375);akt‑1(ok525),* BR6289 *shc‑1(ok198);daf‑18(e1375);akt‑1(ok525),* BR6309 *shc‑1(ok198) daf‑16(mu86);daf‑18(e1375);akt‑1(ok525),* FK171 *mek-1(ks54),* BR5240 *mek‑1(ks54);Is[daf‑16::gfp]*, VC8 *jnk-1(gk7)*, BR6000 *jnk‑1(gk7) Is[daf‑16::gfp]*. BR6540 *shc-1(ok198);sgk-1(ok538);Is[daf-16::gfp]*, BR6543 *shc‑1(ok198) glp‑1(e2141);Is[daf‑16::gfp]*. To generate transgenic animals carrying wild type *daf-16::gfp* or *daf-16(4A)::gfp,* the corresponding constructs were injected into wild type, *shc‑1(ok198)* (for allele numbers, see Supplemental Table S3). 20 ng/µl pRF4 *rol-6* (*su1006)* was used as co-injection marker. GFP expression of the transgenic animals was confirmed using fluorescent microscopy.

**Plasmids**

All constructs, if not mentioned otherwise, were generated with the pEGFP‑N1 vector (Clontech). The four mutations (T54A, S240A, T242A and S314A) in *daf-16*(4A) were generated by oligo‑mediated introduction of site-specific mutations (Stratagene). The *daf‑16::gfp* (pBY2822) and *daf‑16(4A)::gfp* (pBY2916) translational fusions were generated by inserting *daf-16* or *daf-16(4A)* (isoform a) cDNA at the *Sma*I*/Age*I sites and 6.4 kb upstream regulatory sequence of *daf‑16a* at the *Eco*47III/*Bgl*II sites. To express *daf‑16(4A*) in the hypodermis (pBY3077), neurons (pBY3075) and intestine (pBY3074), respectively, *dpy‑7*, *unc‑119*, and *ges‑1* promoters were inserted at the *Eco*47III/*Hin*dIII, *Eco*47III/*Bgl*II and *Eco*47III/*Sma*I sites to fuse them to *daf‑16(4A)* cDNA, which was inserted at the *Sma*I/*Age*I sites. To express *daf‑16(4A*) in the musculature (pBY3073), the *myo‑3* promoter was subcloned from pPD114.95 using the *Hin*dIII/*Bam*HI sites, fusing to *daf‑16(4A)* cDNA, which was inserted at the *Age*I site.

**Quantification of brood size**

Single L4 hermaphrodites were transferred on agar plates containing the *E.* *coli* as food and transferred onto new plates every day until the end of their reproductive period. The eggs or larvae on the plates were counted and removed afterwards. The total number of progeny of each fertile hermaphrodite tested is termed brood size. Sterile animals and those have egg-laying defect are censored.

**Antibody staining**

A formaldehyde fixation procedure was used (Protocol obtained from Michael Koelle, Yale) for the whole worm staining

Fixation

Wash worms off plate with M9, spin them down in a clinical centrifuge, and resuspend with dH_2_O to wash out most of the bacteria. Transfer the worms to a microfuge tube with a pasteur pipette, spin 3K for 30 sec. and remove some supernatant to leave 0.5 ml in the tube. Place on ice to chill. Add 0.5 ml cold 2× witches brew (160 mM KCl, 40 mM NaCl, 20 mM Na_2_EGTA,10 mM spermidine HCl, 30 mM Na Pipes (pH 7.4), 50% methanol, add BME frish just for the uasage to 2%), and 20% formaldehyde to a final concentration of 1-4% (1% is typical). Mix well, and freeze in liquid nitrogen and thaw quickly in a 70°C water bath. Incubate at 4°C with occasional agitation for 30 min to overnight (30 min to a couple of hours is typical). Wash the worms twice in Tris-Triton buffer (100 mM Tris‑HCl pH 7.4, 1% Trion X-100, 1 mM EDTA). Incubate in 1 ml 1% ßME/Tris-Triton for 1-2 hours at 37°C rotating. Wash in ~1 ml 1× borate buffer (40× borate buffer: 1 M H_3_BO_3_, 0.5 M NaOH, Na_2_HPO_4_.7H_2_O, pH >9.5, add more NaOH if required). Incubate in 1 ml 10 mM DTT/1× borate buffer for 15 min at room temp. Wash in ~1 ml borate buffer. Incubate in 0.3% H_2_O_2_/1× borate buffer 15 min at room temp. Wash ~1 min in borate buffer and then incubate 15 min in PBST‑B (1× PBS, 0.1% BSA, 0.5 % Triton X100, 5 mM sodium azide, 1 mM EDTA). At this point the worms are stable and can be stored in PBST-B in the refrigerator indefinitely.

Antibody incubation

Transfer a suspension of worms containing the equivalent of ~5µl of packed worms to a 0.5 ml tube, spin and remove as much liquid as possible, and add ~20 µl antibody diluted 1:200 in PBST‑A (Same as PBST-B except 1% BSA). Incubate at room temperature overnight. Wash the worms 4 times for 25 min each on a rotator at room temperature in PBST-B.

Incubate 1-2 hours at room temperature in 20 µl 2nd antibody 1:25 diluted in PBST-A. Wash the worms 4 times for 25 minutes each on a rotator at room temperature in PBST-B. The stained worms can be stored for months at 4°C in the dark.
